# Bose–Einstein condensate soliton qubit states for metrological applications

**DOI:** 10.1038/s41598-021-97971-4

**Published:** 2021-09-29

**Authors:** The Vinh Ngo, Dmitriy V. Tsarev, Ray-Kuang Lee, Alexander P. Alodjants

**Affiliations:** 1 Institute of Advanced Data Transfer Systems, ITMO University, St. Petersburg, Russia 197101; 2 Institute of Photonics Technologies, National Tsing Hua University, Hsinchu, 30013 Taiwan; 3 Physics Division, National Center for Theoretical Sciences, Taipei, 10617 Taiwan; 4 Center for Quantum Technology, Hsinchu, 30013 Taiwan

**Keywords:** Physics, Quantum physics, Quantum metrology

## Abstract

We propose a novel platform for quantum metrology based on qubit states of two Bose–Einstein condensate solitons, optically manipulated, trapped in a double-well potential, and coupled through nonlinear Josephson effect. We describe steady-state solutions in different scenarios and perform a phase space analysis in the terms of population imbalance—phase difference variables to demonstrate macroscopic quantum self-trapping regimes. Schrödinger-cat states, maximally path-entangled (*N*00*N*) states, and macroscopic soliton qubits are predicted and exploited to distinguish the obtained macroscopic states in the framework of binary (non-orthogonal) state discrimination problem. For an arbitrary frequency estimation we have revealed these macroscopic soliton states have a scaling up to the Heisenberg and super-Heisenberg (SH) limits within linear and nonlinear metrology procedures, respectively. The examples and numerical evaluations illustrate experimental feasibility of estimation with SH accuracy of angular frequency between the ground and first excited macroscopic states of the condensate in the presence of moderate losses, which opens new perspectives for current frequency standard technologies.

## Introduction

Nowadays, the formation and interaction of nonlinear collective modes in Kerr-like medium represent an indispensable platform for various practical applications in time and frequency metrology^1,2^, spectroscopy^3,4^, absolute frequency synthesis^5^, and distance ranging^6^. In photonic systems, frequency combs are proposed for these purposes^7^. The combs occur due to the nonlinear mode mixing in special (ring) microcavities, which possess some certain eigenmodes. Notably, bright soliton formation emerges with vital phenomena accompanying micro-comb generation^8^. Physically, such a soliton arises due to the purely nonlinear effect of temporal self-organization pattern occurring in an open (driven-dissipative) photonic system. However, because of the high level of various noises such systems can be hardly explored for purely quantum metrological purposes.

On the other hand, atomic optics, which operates with Bose–Einstein condensates (BECs) at low temperatures, provides a suitable platform for various quantum devices that may be useful for metrology and sensing tasks^9^.

In particular, so-called Bosonic Josephson junction (BJJ) systems, established through two weakly linked and trapped atomic condensates, are at the heart of the current quantum technologies in atomtronics, which considers atom condensates and aims to design (on-chip) quantum devices^10^. Condensates in this case represent low dimensional systems and may be manipulated by magnetic and laser field combinations. For a real-world experiment we can exploit a Feshbach resonance technique to tune the sign and magnitude of the effective atom-atom scattering length^11^. Thus they represent advanced alternative to optical analogues.

The BJJs are intensively discussed and examined both in theory and experiment^12–16^. The quantum properties of the BJJs are also widely studied^17–24^ including spin-squeezing and entanglement phenomena^19,25,26^, as well as the capability of generating *N*00*N*-states^20,21^ to go beyond the standard quantum limit^27^. Physically, the BJJs possess interesting features connected with the interplay between quantum tunneling of the atoms and their nonlinear properties evoked by atom–atom interaction^28,29^.

Recently, nonlinear effects were recognized as the most interesting and promising from a practical point of view in quantum metrology^30^. For instance, atomic BECs pave the way for the nonlinear quantum metrology approach, which permits the super-Heisenberg (SH) scaling, i.e. scaling beyond Heisenberg limit (HL), cf.^31,32^. It was experimentally demonstrated (see^33,34^) that atomic spin-squeezed states improve the metrological parameter, which plays an important role in spectroscopy and quantum metrology of frequency standards^35^. Obviously, a further enhancement of quantum metrological measurements may be achieved by improving the sources of entangled superposition (*N*00*N*-like) states or entangled states optimally adapted to moderate level of losses^36–38^. For these purposes, we propose to use in this work two-soliton states of atomic condensates.

With Kerr-like nonlinearities, solitons naturally emerge from atomic condensates in low dimensions^39–43^. Especially, the bright atomic solitons observed in lithium condensate possessing a negative scattering length^41–43^ are worth noticing. Atomic gap solitons are also observed in condensates with repulsive inter-particle interaction^40^.

Bright atomic solitons represent a promising platform for high precision interferometry due to the enhancement of fringe contrast. In Refs.^44–46^ authors analyse the matter-wave gyroscope based on the Sagnac atomic interferometer with solitons. However, as shown in Ref.^44^, the analysis of the Fisher information and frequency measurement sensitivity parameter requires a delicate approach based on application of various quantum methods combination in the case of bright solitons interferometry.

Based on soliton modes, we recently proposed the quantum soliton Josephson junction (SJJ) device with the novel concept to improve the quantum properties of the effectively coupled two-mode system^31,32,47,48^. The SJJ-device consists of two weakly-coupled condensates trapped in a double-well potential and elongated in one dimension. BECs with such a geometry were studied in Ref.^49^. We demonstrated that quantum solitons may be explored for the improvement of phase measurement and estimation up to the HL and beyond^47^. In the framework of nonlinear quantum metrology, we also showed that solitons permit a SH scaling $\propto N^{-5/2}$) even with coherent probes^32^. On the other hand, steady-states of coupled solitons can be useful for effective formation of Schrödinger-cat (SC) superposition state and maximally path-entangled *N*00*N*-states, which can be applied for the phase estimation purposes^48^. It is important that such superposition states arise only for soliton-shape condensate wave functions and occur due to the existence of certain steady-states in the phase difference—population imbalance phase plane^32^.

Remarkably, macroscopic states, like SC-states, play an essential role for current information and metrology^50^. In quantum optics, various strategies are proposed for the creation of photonic SC-states and relevant (continuous variable) macroscopic qubits^51–53^. Special (projective) measurement and detection techniques are also important here^54–56^. The condensate environment, dealing with mater waves, is potentially promising for macroscopic qubits implementation due to the minimally accessible thermal noises it provides^57–59^.

In this work, we propose two-soliton superposition states as macroscopic qubits. The interaction between these solitons comes from the nonlinear mode mixing in an atomic condensate trapped in a double-well potential. In particular, we demonstrate the SC-states formation and their implementation for arbitrary phase measurement prior HL and beyond. As we show further, this accuracy is due to the essentially nonlinear behavior of the solitons relative phase. Since SC-states are non-orthogonal states, a special measurement procedure is applied by so-called sigma operators, as it enables us to estimate the unknown phase parameter^60^. On the other hand, our approach can be also useful in the framework of discrimination of binary coherent (non-orthogonal) states in quantum information and communication^61,62^. The non-orthogonality of these states leads to so-called Helstrom bound for the quantum error probability that simply indicates the impossibility for a receiver to identify the transmitted state without some errors^63,64^. In quantum metrology, by means of various regimes of condensate soliton interaction, we deal with a set of quantum states, which may be prepared before the measurement. Our results show that these SC-states approach the soliton *N*00*N*-states to minimize the quantum error probability.

## Two-soliton model

### Coupled-mode theory approach

We start with the mean-field description of coupled mode ﻿theory approach to an elongated BEC trapped in $V=V_H+V(x)$ potential, where $V_H$ is a 3D harmonic trapping potential; while *V*(*x*) is responsible for the double-well confinement in one (*X*) dimension^48^. The (rescaled) condensate wave function (mean field amplitude) $\Psi (x)$ obeys the familiar 1D Gross–Pitaevskii equation (GPE), cf.^49^:

1 $$\begin{aligned} i\frac{\partial }{\partial t}\Psi = -\frac{1}{2}\frac{\partial ^2}{\partial x^2}\Psi - uN\left| \Psi \right| ^2\Psi + V(x)\Psi , \end{aligned}$$

where $u=4\pi |a_{sc}|/a_{\perp }$ characterizes a Kerr-like (focusing) nonlinearity, $a_{sc}<0$ is the s-wave scattering length that appears due to atom-atom scattering in Born-approximation, $a_{\perp }=\sqrt{\hbar /m\omega _{\perp }}$ characterizes the trap scale, and *m* is the particle mass. To be more specific, we only consider condensates possessing a negative scattering length. In Eq. (1) we also propose rescaled (dimension-less) spatial and time variables, which are $x,y,z \rightarrow x/a_{\perp },y/a_{\perp },z/a_{\perp }$, and $t \rightarrow \omega _{\perp } t$, cf.^32,47,49^.

The nonlinear coupled-mode theory admits a solution of Eq. (1) that simply represents a quantum-mechanical superposition

2 $$\begin{aligned} \Psi (x,t)=\Psi _1(x,t)+\Psi _2(x,t), \end{aligned}$$

where the wave functions $\Psi _{1}(x)$ and $\Psi _{2}(x)$ characterize the condensate in two wells. For weakly interacting atoms one can assume that

3 $$\begin{aligned} \Psi _{1,2}(x,t)=C_{1,2}(t)\Phi _{1,2}(x)e^{-i\beta _{1,2}t}, \end{aligned}$$

where $\Phi _1(x)$ and $\Phi _2(x)$ are ground- and first-order excited states, with the corresponding wave functions possessing energies $\beta _1$ and $\beta _2$, respectively; $C_{1}(t)$ and $C_{2}(t)$ are time-dependent functions. If the particle number is not too large, Eq. (3) may be integrated in spatial dimension, leaving only two condensate variables $C_{1,2}(t)$^29^. In particular, $\Phi _1(x)$ and $\Phi _2(x)$ may be time-independent Gaussian-shape wave functions obeying different symmetry. Practically, this two-mode approximation is valid for the condensates of several hundreds of particles^65^. The condensate in this limit is effectively described by two macroscopically populated modes as a result.

### Quantization of coupled solitons

**Figure 1:**
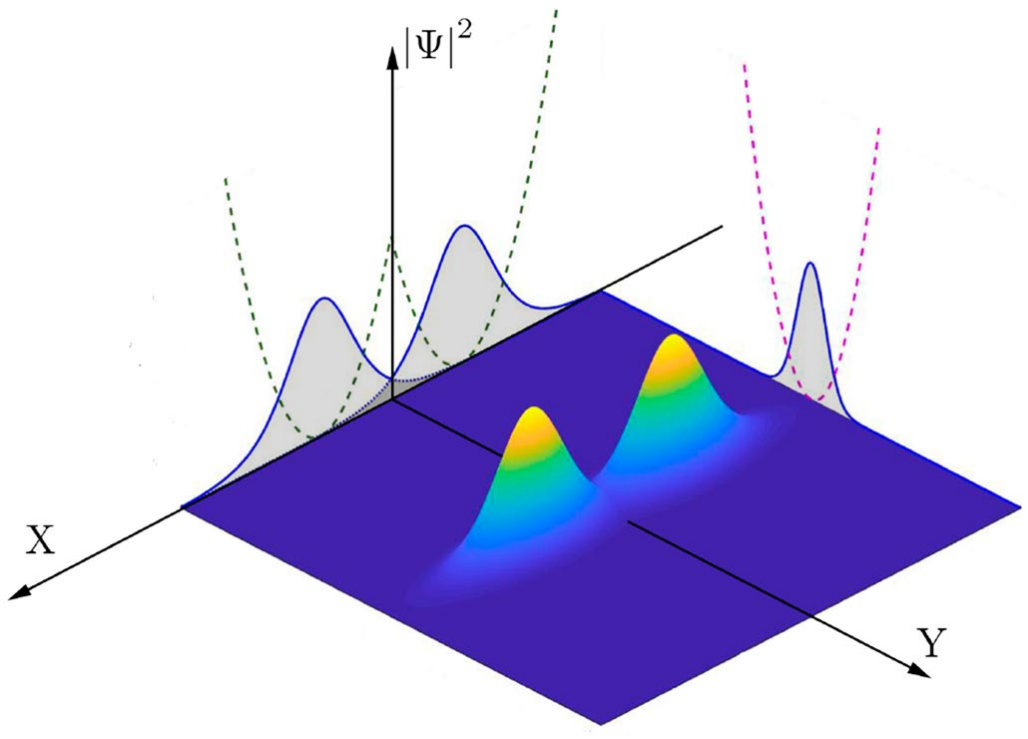
Sketch of the probability density distribution $|\Psi |^2$ versus spatial coordinates *X* and *Y*, as a 2D projection of the 3D coupled condensates trapped in a double-well (dashed green curve) and harmonic (dashed magenda curve) potentials, respectively. Shadow regions display 1D condensate wave packets projections; they represent a secant-shape in *X*-direction and Gaussian-shape in the transverse directions.

The sketch in Fig. 1 explains the two-soliton system described in our work. If trapping potential *V*(*x*) is weak enough and the interaction among condensated particles is not so weak, the ansatz solution (3) is no longer suitable. For condensates with a negative scattering length, a bright soliton solution is admitted for $\Psi _{1,2}(x,t)$ in Eq. (2). In fact, in this case one can speak about two-soliton solution problem, which is well known in classical theory of solitons^66^.

In quantum theory, instead of Eq. (2), we deal with a bosonic field operator ${\hat{a}}(x,t)\propto {\hat{a}}_1+{\hat{a}}_2$, where ${\hat{a}}_{1,2}\equiv {\hat{a}}_{{1,2}}(x,t)$ are field operators corresponding to mean-field amplitudes $\Psi _{1,2}(x,t)$. We assume that experimental conditions allow the formation of atomic bright solitons in each of the wells. In particular, these conditions may be realized by means of manipulation with weakly trapping potential *V*(*x*). Experimentally, this manipulation may be performed by a dipole trap and laser field.

Then, considering linear superposition state, one can write down the total Hamiltonian ${\hat{H}}$ for two BEC solitons in the second quantization form as

4 $$\begin{aligned} {\hat{H}} = \int _{-\infty }^\infty \sum _{j=1}^2\left( {\hat{a}}_j^\dag \left( -\frac{1}{2}\frac{\partial ^2}{\partial x^2}\right) {\hat{a}}_{j}dx\right) - \frac{u}{2}\int _{-\infty }^\infty \Big ({\hat{a}}_1^\dag +{\hat{a}}_2^\dag \Big )^2\Big ({\hat{a}}_1+{\hat{a}}_2\Big )^2dx. \end{aligned}$$

The annihilation (creation) operators of bosonic fields, denoted as ${\hat{a}}_{j}$ (${{\hat{a}}^\dag _{j}}$) with $j=1,2$, obey the commutation relations:

5 $$\begin{aligned} {[}{\hat{a}}_i(x), {{\hat{a}}^\dag _{j}(x')}] = \delta (x-x')\,\delta _{ij}; \quad i,j =1,2. \end{aligned}$$

In the Hartree approximation for a large particle number, $N>>1$, one can assume that the quantum *N*-particle two-soliton state is the product of *N* two-soliton states and can be written as^67–69^

6 $$\begin{aligned} \left| \Psi _N\right\rangle = \frac{1}{\sqrt{N!}}\left[ \int _{-\infty }^\infty \left( \Psi _1(x,t){\hat{a}}_1^\dag e^{-i\beta _1t} + \Psi _2(x,t){\hat{a}}_2^\dag e^{-i\beta _2t}\right) dx\right] ^N\left| 0\right\rangle , \end{aligned}$$

where $\Psi _j(x,t)$ is the unknown wave functions, and $|0\rangle \equiv |0\rangle _1 |0\rangle _2$ denotes a two-mode vacuum state. The state given in Eq. (6) is normalized as $\left\langle \Psi _N\big |\Psi _N\right\rangle = 1$, and the bosonic field-operators ${\hat{a}}_{j}$ act on it as

7 $$\begin{aligned} {\hat{a}}_j\left| \Psi _N\right\rangle = \sqrt{N}\Psi _j(x,t)e^{-i\beta _jt}\left| \Psi _{N-1}\right\rangle . \end{aligned}$$

Applying variational field theory approach based on the ansatz $\Psi _j(x,t)$, one can obtain the Lagrangian density in the form:

8 $$\begin{aligned} L_0 = \frac{1}{2} \sum _{j=1}^2\left( i\left[ \Psi _j^*{\dot{\Psi }}_j - {\dot{\Psi }}_j^*\Psi _j\right] - \left| \frac{\partial \Psi _j}{\partial x}\right| ^2\right) + \frac{uN}{2}\left( \Psi _1^*e^{i\beta _1t} +\Psi _2^*e^{i\beta _2t}\right) ^2\left( \Psi _1e^{-i\beta _1t}+\Psi _2e^{-i\beta _2t}\right) ^2, \end{aligned}$$

where we suppose $N-1\approx N$ and omit common term *N*.

Noteworthy, from Eq. (8), one can obtain the coupled GPEs for $\Psi _j$-functions as

9 $$\begin{aligned} i\frac{\partial }{\partial t}\Psi _1= & {} -\frac{1}{2}\frac{\partial ^2}{\partial x^2}\Psi _1 - uN\left( \left| \Psi _1\right| ^2 + 2\left| \Psi _2\right| ^2\right) \Psi _1\nonumber \\&- uN\left( \left| \Psi _2\right| ^2 + 2\left| \Psi _1\right| ^2\right) \Psi _2e^{-i\Omega t} - uN\Psi _1^*\Psi _2^2e^{-2i\Omega t} - uN\Psi _2^*\Psi _1^2e^{i\Omega t}, \end{aligned}$$

10 $$\begin{aligned} i\frac{\partial }{\partial t}\Psi _2= & {} -\frac{1}{2}\frac{\partial ^2}{\partial x^2}\Psi _2 - uN\left( \left| \Psi _2\right| ^2 + 2\left| \Psi _1\right| ^2\right) \Psi _2\nonumber \\&- uN\left( \left| \Psi _1\right| ^2 + 2\left| \Psi _2\right| ^2\right) \Psi _1e^{i\Omega t} - uN\Psi _2^*\Psi _1^2e^{2i\Omega t} - uN\Psi _1^*\Psi _2^2e^{-i\Omega t}, \end{aligned}$$

where $\Omega =\beta _2-\beta _1$ is the energy (frequency) spacing.

The set of Eqs. (9) and (10) leads to the known problem for transitions between two lowest self-trapped states of condensates in the nonlinear coupled mode approach if we account Eq. (3) for the representation of condensate wave functions $\Psi _j(x,t)$^28,29^.

On the other hand, Eqs. (9) and (10) can be recognized in the framework of soliton interaction problem that may be solved by means of perturbation theory for solitons^66^. In particular, in accordance with Karpman’s approach we can find in Eq. (9) and (10) the terms proportional to $\epsilon _{jk}=\Psi _j^*\Psi _k^2 + 2|\Psi _j|^2\Psi _k$, $j,k=1,2$, $j\ne k$, as perturbations for two fundamental bright soliton solutions. Physically, $\epsilon _{jk}$ implies the nonlinear Josephson coupling between the solitons.

In this work we establish a variational approach for the solution of Eqs. (9) and (10), cf.^32^. For the weakly coupled condensate states, i.e. for $\epsilon _{jk}\simeq 0$, the set of Eqs. (9) and (10) can be reduced to two independent GPEs:

11 $$\begin{aligned} i\frac{\partial }{\partial t}\Psi _j = -\frac{1}{2}\frac{\partial ^2}{\partial x^2}\Psi _j - uN\left| \Psi _j\right| ^2\Psi _j, \end{aligned}$$

which possess bright (non-moving) soliton solutions

12 $$\begin{aligned} {\Psi _j(x,t)} = \frac{N_j}{2}\sqrt{\frac{u}{N}}{{\,\text{ {sech}}\,}}\left[ \frac{uN_j}{2}x\right] e^{i\frac{u^2N_j^2}{8}t}. \end{aligned}$$

In the case of $\epsilon _{jk}\ne 0$ and non-zero inter-soliton distance $\delta$, we examine ansatzes for $\Psi _j(x,t)$ in the form

13 $$\begin{aligned} \Psi _1(x,t)= & {} \frac{N_1}{2}\sqrt{\frac{u}{N}}{{\,\text{ {sech}}\,}}\left[ \frac{uN_1}{2}\left( x - \delta \right) \right] e^{i\theta _1}, \end{aligned}$$

14 $$\begin{aligned} \Psi _2(x,t)= & {} \frac{N_2}{2}\sqrt{\frac{u}{N}}{{\,\text{ {sech}}\,}}\left[ \frac{uN_2}{2}\left( x + \delta \right) \right] e^{i\theta _2}. \end{aligned}$$

In particular, our approach presumes the existence of two well distinguished solitons (separated by the small distance $\delta$, with the shape preserved) interacting through dynamical variation of the particle numbers, $N_j\equiv N_j(t)$, and phases, $\theta _j\equiv \theta _j(t)$, which occurs in the presence of weak coupling between the solitons. In other words, $N_j$ and $\theta _j$ should be considered as time-dependent (variational) parameters.

By substituting Eqs. (13) and (14) into (8) we obtain (up to the constant factor and term)

15 $$\begin{aligned} L = \int _{-\infty }^\infty L_0 dx\simeq - z{\dot{\theta }} + \Lambda z^2 + \frac{\Lambda }{2}\left( 1 - z^2\right) ^2I(z,\Delta )\left( \cos [2\Theta ]+2\right) + \Lambda \left( 1 - z^2\right) J(z,\Delta )\cos [\Theta ], \end{aligned}$$

where $z=(N_2-N_1)/N$ ($N_{1,2}=\frac{N}{2}(1\mp z)$) is the particle number population imbalance; $\Theta =\theta _2-\theta _1-(\beta _2-\beta _1)t \equiv \theta - \Omega t$ is an effective time-dependent phase-shift between the solitons.

Physically, $\Omega$ is an angular frequency spacing between the ground and first excited macroscopic states of the condensate; it represents a vital (measured) parameter for metrological purposes in this work. In Eq. (15), we also introduce the notation $\Lambda =N^2u^2/16$ and define the functionals

16 $$\begin{aligned} I&\equiv I(z,\Delta ) = \int _{-\infty }^\infty {{\,\text{ {sech}}\,}}^2\left[ \left( 1 -z\right) \left( x-\Delta \right) \right] {{\,\text{ {sech}}\,}}^2\left[ \left( 1+z\right) \left( x+\Delta \right) \right] dx, \end{aligned}$$

17 $$\begin{aligned} J&\equiv J(z,\Delta ) = \sum _{s=\pm 1}\left( \int _{-\infty }^\infty \left( 1 +sz\right) ^2{{\,\text{ {sech}}\,}}^3\left[ \left( 1+sz\right) \left( x+s\Delta \right) \right] {{\,\text{ {sech}}\,}}\left[ \left( 1-sz\right) \left( x-s\Delta \right) \right] \right) , \end{aligned}$$

where $\Delta \equiv ~\frac{Nu}{4}\delta$ is a normalized distance between solitons.

Finally, by using Eq. (15) for the population imbalance and phase-shift difference, *z* and $\Theta$, we obtain the set of equations

18 $$\begin{aligned} {\dot{z}}= & {} \left( 1-z^2\right) \left\{ \left( 1-z^2\right) I\sin [2\Theta ] + J\sin [\Theta ]\right\} , \end{aligned}$$

19 $$\begin{aligned} {\dot{\Theta }}= & {} - \frac{\Omega }{\Lambda } + 2z + \frac{{\text {d}}}{{\text {d}}z}\left\{ \frac{1}{2}\left( 1-z^2\right) ^2I\left( \cos [2\Theta ]+2\right) + \left( 1-z^2\right) J\cos [\Theta ]\right\} , \end{aligned}$$

where dots denote the derivatives with respect to the renormalized time $\tau =\Lambda t$.

In contrast to the problem with coupled Gaussian-shape condensates, the solutions of Eqs. (18) and (19) crucially depend on the features of governing functionals $I(z,\Delta )$ and $J(z,\Delta )$, cf.^28,29^. In Supplementary Material we represent some analytical approximations for $I(z,\Delta )$ and $J(z,\Delta )$, in order to give a clear illustration.

## Steady-state (SS) solutions

### Steady-state solution for $z^2=1$

The steady-state (SS) solutions of Eqs. (18) and (19) play a crucial role for metrological purposes with coupled solitons^47^. We start from the SS solution $z^2=1$ of Eq. (18) by setting the time-derivatives to zero. As seen from Eqs. (16) and (17), in the limit of maximal population imbalance, $z^2=1$, *I* and *J* are independent on $\Delta$ and approach

20 $$\begin{aligned} I(z,\Delta )= & {} 1, \end{aligned}$$

21 $$\begin{aligned} J(z,\Delta )= & {} \pi . \end{aligned}$$

Substituting $z^2=1$ and Eqs. (20) and (21) into Eq. (19), we obtain

22 $$\begin{aligned} z^2= & {} 1, \end{aligned}$$

23 $$\begin{aligned} \Theta= & {} \arccos \left[ \frac{2\Lambda - {{\,\text{ {sign}}\,}}[z]\Omega }{2\pi \Lambda }\right]. \end{aligned}$$

Notably, in the quantum domain the SS solutions shown in Eqs. (22) and (23) admit the existence of quantum states with maximal population imbalance $z = \pm 1$ and phase difference. The latter depends on the frequency spacing $\Omega$, which is the object of precise measurement with maximally path-entangled *N*00*N*-states in this paper.

Below we perform the analysis of the SS solutions of Eqs. (18) and (19) in two limiting cases $\Omega \ne 0$, $\Delta \simeq 0$ and $\Omega \simeq 0$, $\Delta \ne 0$.

### SS solutions for $\Theta =0,\pi$ and $\Delta \simeq 0$

To find the SS solutions we rewrite Eq. (19) as

24 $$\begin{aligned} \frac{\Omega }{\Lambda } = 2z - 6z\left( 1-z^2\right) I + \frac{3}{2}\left( 1-z^2\right) ^2\frac{\partial I}{\partial z} - 2zJ + \left( 1-z^2\right) \frac{\partial J}{\partial z}, \end{aligned}$$

for $$\Theta =0$$ and

25 $$\begin{aligned} \frac{\Omega }{\Lambda } = 2z - 6z\left( 1-z^2\right) I + \frac{3}{2}\left( 1-z^2\right) ^2\frac{\partial I}{\partial z} + 2zJ - \left( 1-z^2\right) \frac{\partial J}{\partial z}, \end{aligned}$$

for $$\Theta =\pi$$, respectively.

In Supplementary Material we represent a polynomial approximation for *I*, *J* functionals given in Eqs. (16) and (17). Since the equations obtained from Eqs. (24) and (25) are quite cumbersome, here we just briefly analyze the results.

In the limit of closely spaced solitons and $\Theta =0$, the population imbalance *z* at equilibrium depends only on $\Omega$ and obeys

26 $$\begin{aligned} \frac{\Omega }{\Lambda } = 1.2 z^7 - 8 z^5 + 15 z^3 - 12.5 z. \end{aligned}$$

Similarly, for fixed soliton phase difference $\Theta =\pi$ we have

27 $$\begin{aligned} \frac{\Omega }{\Lambda } = 1.2 z^7 - 3.2 z^5 + 12.3 z^3 - 2 z. \end{aligned}$$

**Figure 2:**
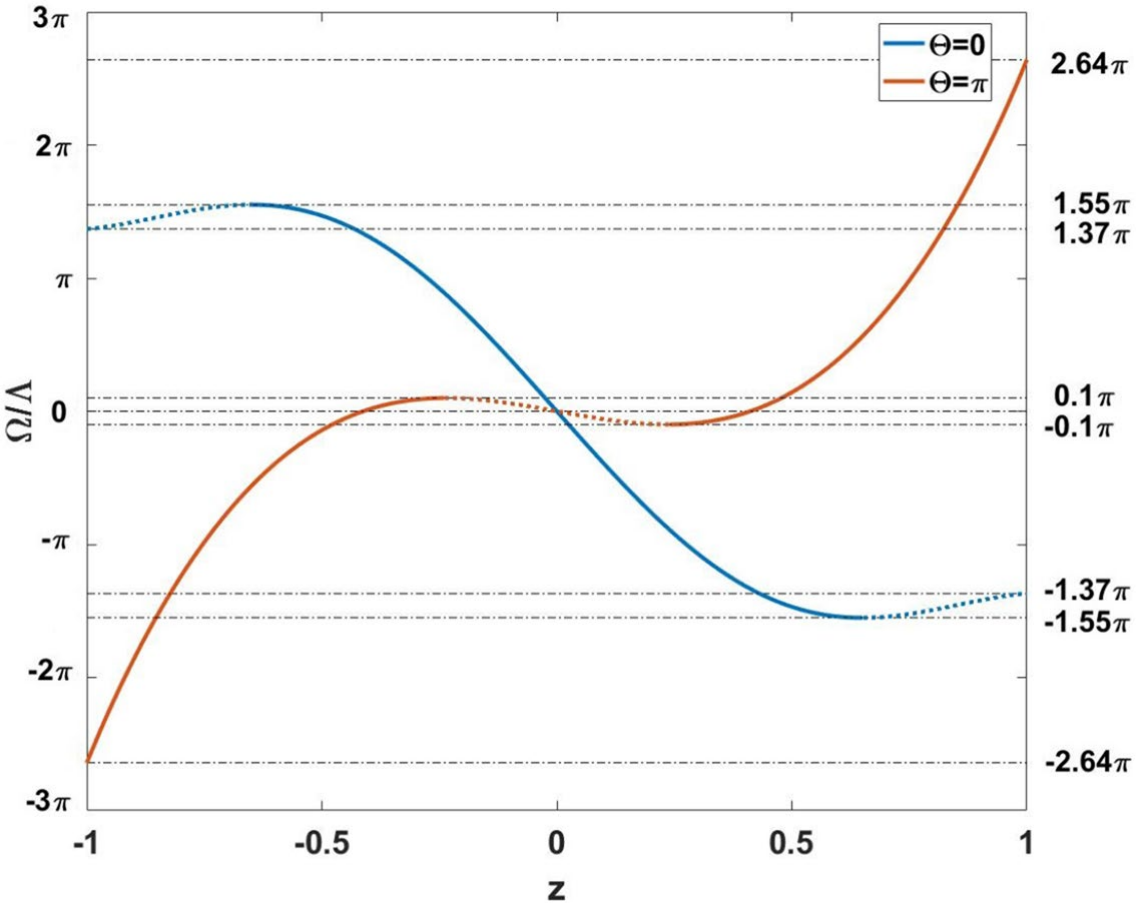
Normalized frequency spacing $\Omega /\Lambda$ (dashed lines) versus reduced population imbalance *z* for Eq. (26) (blue line) and Eq. (27) (red line), respectively. Dashed regions correspond to unstable solutions.

We plot the graphical solutions of Eqs. (26) and (27) in Fig. 2; the blue and red curves characterize the right parts of Eqs. (26) and (27), respectively. The straight lines in Fig. 2 correspond to different values of the $\Omega /\Lambda$ ratio. These lines cross the curves in the points indicating the solutions of Eqs. (26) and (27). Notice that the solid blue and red curves denote the values of $\Omega /\Lambda$ and *z* corresponding to the stable SS solutions; while the dotted ones describe parametrically unstable solutions. As seen from Fig. 2, at phase difference $\Theta =0$ there exists one stable SS solution for any $z\in [-0.7;0.7]$ and only unstable solutions for $|z|>0.7$. At $|\Omega /\Lambda |>1.55\pi$, no SS solutions exist.

On the other hand, at $\Theta =\pi$ there exists a tiny region $-0.1\pi \le \Omega /\Lambda \le 0.1\pi$ possessing two SS solutions simultaneously. One stable SS solution exists within the domain $0.1\pi <|\Omega /\Lambda |\le 2.64\pi$.

### SS solutions for $\Theta =0,\pi$ and $\Omega \simeq 0$

At $\Omega =0$ Eqs. (26) and (27) admit the SS solutions, which look like:

28 $$\begin{aligned}&z=0,\Theta =0, \end{aligned}$$

29 $$\begin{aligned}&z=0,\Theta =\pi,\end{aligned}$$

30 $$\begin{aligned}&z^2\approx 0.17, \Theta =\pi . \end{aligned}$$

As seen from Eq. (29), at relative phase $\Theta =\pi$ Eq. (25) possesses three solutions: a parametrically unstable solution occurs at $z=0$ and two degenerate SS solutions appear for $z=\pm z_0$. Here, $z_0$ varies from 0.41 at $\Delta \approx 0$ to 0.64 at $\Delta \approx 2.8$ for non-zero soliton inter-distance, respectively. For $\Delta > 2.8$ these SS solutions do not exist.

**Figure 3:**
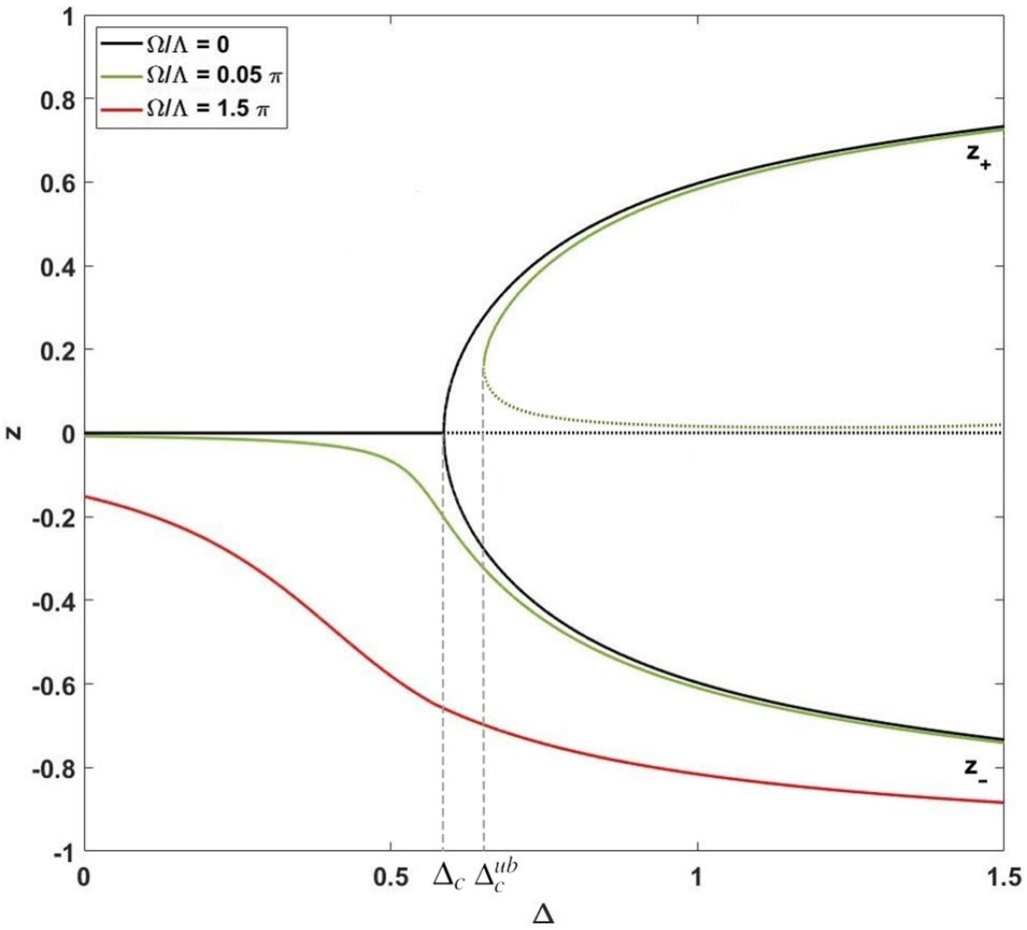
Population imbalance *z* versus $\Delta$ for $\Theta =0$ and different $\Omega$. The solid curves denote SS solutions of Eq. (24) and the dotted curves represent (parametrically) unstable solutions. $\Delta _c = 0.5867$ and $\Delta _c^{ub} = 0.647$ are critical distances between the solitons correspondent to bifurcations occurring for curves $\Omega /\Lambda = 0$ and $\Omega /\Lambda = 0.05\pi$, respectively.

In Fig. 3 we represent a more general analysis of SS solutions for $\Theta =0$ as functions of inter-soliton distance $\Delta$ for different $\Omega$. For that we exploit the sixth-order polynomial approximation, see Supplementary Material.

Notably, the dependence in Fig. 3 is similar to the one obtained with the Lipkin–Meshkov–Glick (LMG) model, see e.g.^70^. The LMG model exhibits remarkable features including quantum phase transition and maximally entangled state formation, see e.g.^71–75^. In our work, the distance between solitons plays a key role in this case. In particular, at $\Omega \simeq 0$ there exists one solution at $z=0$, stable at $\Delta \le \Delta _c\approx 0.5867$. For $\Delta >\Delta _c$ this solution becomes parametrically unstable. On the other hand, for $\Delta >\Delta _c$ Eq. (24) possess the degenerate SS solutions similar to the ones at $\Theta =\pi$. The bifurcation for population imbalance *z* occurs at $\Delta =\Delta _c$; in Fig. 3 the $z_+$ (upper,positive) and $z_-$ (lower, negative) branches characterize this bifurcation. In the vicinity of $\Delta _c$ we can consider $z_\pm =\pm z_0$, where

31 $$\begin{aligned} z_0=1.2\sqrt{\Delta - \Delta _c}. \end{aligned}$$

At $\Omega \ne 0$, the behavior of SS solutions become complicated with respect to the distance $\Delta$—see the green curves in Fig. 3. The solid curves correspond to SS solutions for different $\Delta$, while the dotted ones describe the unstable solutions. As clearly seen from Fig. 3, for $\left| \Omega \right| >0$ there is no bifurcation for population imbalance *z* and two stationary solution branches $z_{\pm }$ occur with $|z_-|>|z_+|$. Notice, for $\Omega >0$ there exists another critical point $\Delta _c^{ub}>\Delta _c$, where the upper branch $z_+$ of SS solution appears. The numerical calculation for $\Omega /\Lambda =0.05\pi$ in Fig. 3 gives $\Delta _c^{ub}\approx 0.647$ or $\Delta _- \approx 0.06$ in (35). For these parameters $z_+ \approx 0.2$ and $z_- \approx -0.3$. On the other hand, at a relatively large values of parameter $\Omega /\Lambda$, only one SS solution exists - see the red curve in Fig. 3.

## Mean-field dynamics

### Small amplitude oscillations

We start our analysis here from small amplitude oscillations close to SS solutions given in Eqs. (28)–(30). For that we linearize Eqs. (18) and (19) in the vicinity of the solution in Eqs. (28) and (30), assuming $0\le \Delta <0.6$ and $\Omega {/\Lambda }<<1$. The first assumption allows us to use the approximation of *I*, *J*-functionals by the fourth-degree polynomial, see Supplementary Material.

For zero-phase oscillations, i.e. for $\Theta \approx 0$ ($\cos \left[ \Theta \right] \approx 1$, $\sin \left[ \Theta \right] \approx \Theta$), from Eqs. (18) and (19) we obtain

32 $$\begin{aligned} \ddot{z} + \omega _0^2(\Delta )z = f_0(\Delta )\frac{\Omega }{{\Lambda }}, \end{aligned}$$

with the solution

33 $$\begin{aligned} z(\tau ) = A\cos \left[ \omega _0\tau \right] - \frac{\Omega }{{\Lambda }}\frac{f_0}{\omega _{0}^2}, \end{aligned}$$

where *A* and $\omega _0(\Delta )=13.4\sqrt{0.37-\Delta ^2 - 0.25\Delta }$ are the amplitude and angular frequency of oscillations, respectively.

Notice, here in (33) and thereafter all frequencies $\omega _j$ ($j=0, \pi , ST$) characterizing small amplitude oscillations are given in $\Lambda ^{-1}$ units due to the time renormalization $\tau = \Lambda t$ performed earlier. The last term in Eq. (33) with $f_0(\Delta )=5.36-0.8\Delta -4.22\Delta ^2$ plays a role of constant “external downward displacement force” that vanishes at $\Omega \simeq 0$. Notably, at $\Delta >0.5$, the oscillations become anharmonic and $z(\tau )$ diverges at $\Delta >0.5867$. For $\Delta =0$ the frequency of oscillations approaches $\omega _0 \approx 8.15$, that agrees with the numerical solution of Eqs. (18) and (19).

At $\Delta =\Delta _c\simeq 0.5867$ SS solution given in Eqs. (28) splits into two degenerate solutions with $z=\pm z_0$ and $z_0$ determined by Eq. (31), see Fig. 3. Near these points the equation, similar to Eq. (32), has a form

34 $$\begin{aligned} \ddot{z} + {\omega _{ST}^2}z = - 18\Delta _-\sqrt{\Delta _-} - \frac{\Omega }{{\Lambda }}f(\Delta _-) \end{aligned}$$

that implies a solution

35 $$\begin{aligned} z(\tau ) = \pm \left( 1.2 - \frac{18\Delta _-}{{\omega _{ST}^2}}\right) \sqrt{\Delta _-} + A\cos [{\omega _{ST}}\tau ] - \frac{\Omega }{{\Lambda }}\frac{f(\Delta _-)}{{\omega _{ST}^2}}, \end{aligned}$$

where $\Delta _-\equiv \Delta -\Delta _c$, ${\omega _{ST}} = 14.53\sqrt{\Delta _{-} - 4.48\Delta _-^2 + 17.8\Delta _-^3 - 53.5\Delta _-^4}$ is the angular frequency of oscillations, and $f = 3.4 - 7.26\Delta _- + 11\Delta _-^2$ is the “external” force. A relative error for Eq. (35) is less than 5%.

In the vicinity of SS points determined by Eq. (30), we obtain $\pi$-phase oscillations characterized by

36 $$\begin{aligned} z(\tau ) = \pm z_0 + A\cos \left[ \omega _{\pi }\tau \right] + \frac{\Omega }{{\Lambda }}\frac{f_{\pi }}{\omega _{\pi }^2}, \end{aligned}$$

with $\omega _{\pi } = \sqrt{2-0.9\Delta ^2 - 0.3\Delta }$, $f_{\pi } = 0.1\left( \Delta ^2 + 0.38\Delta + 5.5\right)$, and $z_0$ determined in Eq. (30). For $\Omega \simeq 0$ and $\Delta =0$, the angular frequency is $\omega _{\pi } \approx 1.42$, which is much smaller than that in the zero-phase regime.

**Figure 4:**
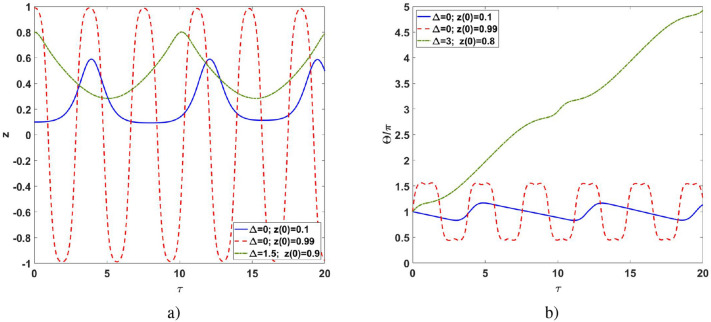
(**a**) The population imbalance $z(\tau )$ and (**b**) effective phase difference $\Theta (\tau )$ versus reduced time $\tau$ for $\Theta (0)=\pi$.

The analysis of Eqs. (18) and (19) in the vicinity of Eq. (29) reveals that this solution is parametrically unstable, and highly nonlinear behavior is expected. Indeed, a direct numerical simulation demonstrates anharmonic dynamics plotted in Fig. 4. For $0<|z|<0.5$ the nonlinear regime of self-trapping is observed,; while it turns into nonlinear oscillations at $|z|>0.5$.

The analysis of SS solution (22) and (23) reveals a strong sensitivity to *z*-perturbation, when condition $z^2=1$ is violated, the high-amplitude nonlinear oscillations occur. On the other hand, solution (22) and (23) is robust to phase-perturbations, which is an important property for metrology.

### Large separation limit, $\Delta>>1$

For a very large distance $\Delta$ between the solitons, i.e., $\Delta>>1$, the atom tunneling between them vanishes, and the solitons become independent. Strictly speaking, in the limit of $\Delta \rightarrow \infty$ the functionals $I,J\rightarrow 0$, and Eqs.  (18) and (19) look like

37 $$\begin{aligned} {\dot{z}}= & {} 0, \end{aligned}$$

38 $$\begin{aligned} {\dot{\Theta }}= & {} - \frac{\Omega }{{\Lambda }} + 2z, \end{aligned}$$

i.e., the population imbalance is a constant in time and the running-phase regime establishes.

For large but finite $\Delta$, SS solution having $z=\pm z_0$ with $z_0\rightarrow 1$ exists for the zero-phase regime, $\Theta =0$; for example, for $\Delta =10$ the SS population imbalance is $z_0\approx 0.96$.

### Phase-space analysis

**Figure 5:**
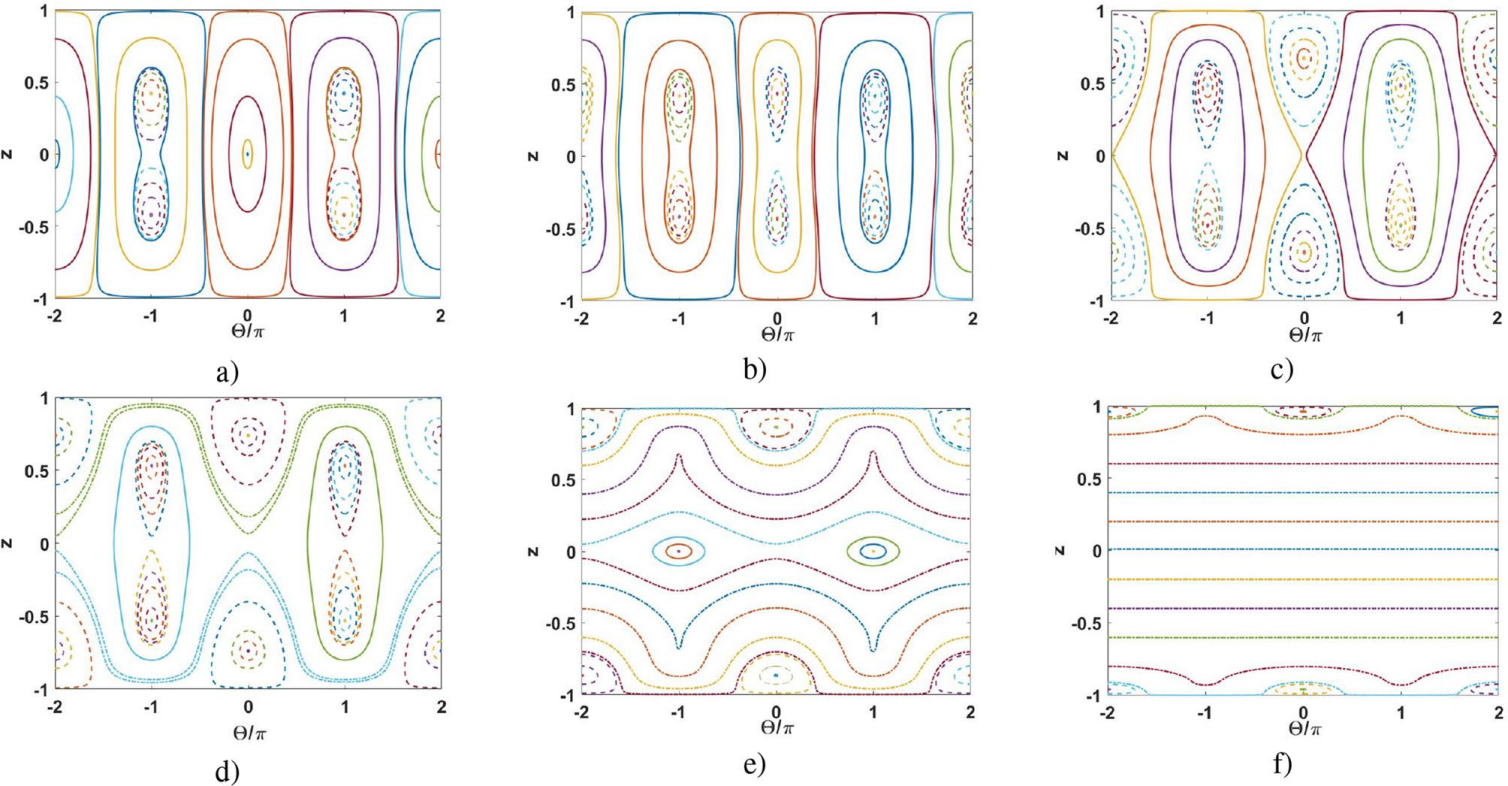
Phase plane $z-\Theta$ at $\Omega =0$ and for (**a**) $\Delta = 0$; (**b**) $\Delta = 0.75$; (**c**) $\Delta = 1.2$; (**d**) $\Delta = 1.5$; (**e**) $\Delta = 3.0$; (**f**) $\Delta = 10$.

**Figure 6:**
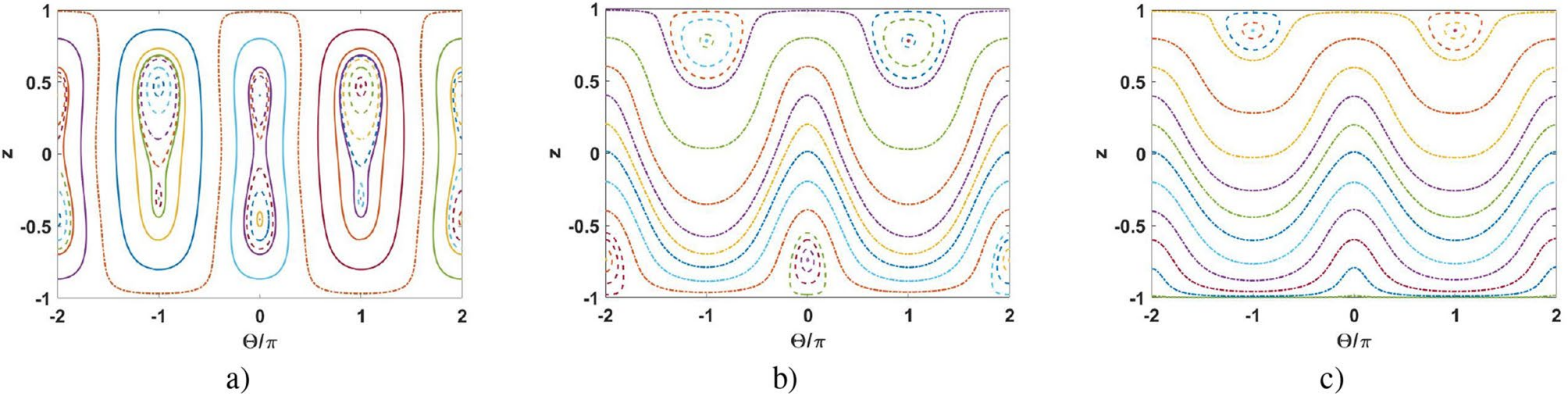
Phase plane $z-\Theta$ at $\Delta = 0.75$ and for (**a**) $\Omega {/\Lambda } = 0.05\pi$; (**b**) $\Omega {/\Lambda } = \pi$; (**c**) $\Omega {/\Lambda } = 1.5\pi$.

The dynamical behavior of the coupled soliton system can be generalized in terms of a phase portrait of two dynamical variables *z* and $\Theta$, as shown in Figs. 5 and 6.

In Fig. 5 we represent $z-\Theta$ phase-plane for $\Omega =0$ and for different (increasing) values of distance $\Delta$. We distinguish three different dynamic regimes. The solid curves correspond to the oscillation regime when $z(\tau )$ and $\Theta (\tau )$ are some periodic functions of normalized time, see Eq. (33) and the red curve in Fig. 4. The dashed curves in Fig. 5 indicate the self-trapping regime when $z(\tau )$ is periodic and the sign of *z* does not change, see Eq. (36) and the blue curve in Fig. 4. Physically, this is the macroscopic quantum self-trapping (MQST) regime characterized by a nonzero average population imbalance when the most of the particles are “trapped” within one of the solitons. At the same time, the behavior of phase $\Theta (\tau )$ may be quite complicated but periodic in time. On the other hand, for the running-phase regime depicted by the dashed-dotted curves, $\Theta (\tau )$ grows infinitely, see the green curve in Fig. 5b. Due to the symmetry that takes place at $\Omega =0$, the running-phase can be achieved only with the MQST regime, see Fig. 5.

As seen from Fig. 5, the central area of nonlinear Rabi-like oscillations between the ground and first excited macroscopic states occur for a relatively small inter-soliton distance $\Delta$ and are inherent to zero-phase oscillations, see Fig. 5a. As discussed before, at $\Delta =\Delta _c\approx 0.5867$ this area splits into two regions characterized by the MQST regimes, Fig. 5b. This splitting occurs due to the bifurcation of population imbalance, see the black curve in Fig. 3. These regions are moving away from each other with growing $\Delta$, see Fig. 5c–f. Notably, the bifurcation effect and MQST states, which are the features of the coupled solitons (Fig. 1) at the zero-phase regime, do not occur for the condensates described by Gaussian states^28,29^.

The phase trajectories inherent to $\pi$-phase region $\frac{\pi }{2}<\Theta <\frac{3\pi }{2}$ stay weakly perturbed until the second critical value $\Delta \approx 2$, when the MQST regime in Fig. 5d changes to Rabi-like oscillations in Fig. 5e, then, approaches the running-phase at $\Delta \approx 6$, see Fig. 5f.

At large enough $\Delta$, the particle tunneling vanishes and the zero-phase MQST domains arise in the vicinity of population imbalance $z=\pm 1$, Fig. 5f. The phase dynamics corresponds to the running-phase regime with $z = {{\,\text{ {const}}\,}}$, see Fig. 5f and Eqs.  (37) and (38).

For non-zero $\Omega$, the phase portrait becomes asymmetric, see Fig. 6. To elucidate the role of $\Omega$, we study the soliton interaction for a given inter-soliton distance $\Delta =0.75>\Delta _c$ that corresponds to the one after the bifurcation. As seen from Fig. 6a, the phase portrait does not change significantly for small $\Omega {/\Lambda }$, cf. Fig. 5b.

One of the SS solutions for zero and $\pi$-phase regimes disappears with increasing $\Omega {/\Lambda }$; then the running-phase regime establishes, see Fig. 6b. Further increasing of $\Omega {/\Lambda }$ leads to vanishing the SS solution for zero-phase, Fig. 6c.

Thus, phase portraits in Figs. 5 and 6 demonstrate the existence of degenerate SSs for coupled solitons by varying $\Delta$ and $\Omega {/\Lambda }$. Such solutions, as we show below, may be exploited for the macroscopic superposition soliton states formation in the quantum approach.

## Quantum metrology with two-soliton states

### Soliton SC-qubit states

In this Section we demonstrate how two-soliton quantum superposition states may be used for the parameter estimation and measurement procedure. In particular, we explore two degenerate states with population imbalance $z=z_\pm$ for these purposes.

In the mean-field approximation $z_\pm$ correspond to two SS solutions, which specify MQST regimes established in Fig. 5 for phases $\Theta =0$ and $\Theta =\pi$ (see Eq. (30)), respectively. Strictly speaking, values $z_\pm$ for $\Theta =0$ are inherent to the upper and lower branches in Fig. 3 and appear above the critical distance $\Delta _c$ between the solitons, cf. Fig. 5b.

In the quantum domain two degenerate SS solutions $z_\pm$ (occurring simultaneously) determine the existence of macroscopic superposition states, which correspond to SC- or *N*00*N*-states. Here, we specify necessary conditions for these states formation within the Hartree approach, cf.^17,47^.

We generalize SC- and *N*00*N*-states as macroscopic qubit states of the solitons, which we define as

39 $$\begin{aligned} |\pi _0\rangle= & {} c_1|\Phi _1\rangle - c_2|\Phi _2\rangle , \end{aligned}$$

40 $$\begin{aligned} |\pi _1\rangle= & {} c_2|\Phi _1\rangle - c_1|\Phi _2\rangle , \end{aligned}$$

where $|\Phi _1\rangle$ and $|\Phi _2\rangle$ are two macroscopic states representing two “halves” of SC-, or *N*00*N*-states. Notice, operators ${\hat{\Pi }}_i= |\pi _i\rangle \langle \pi _i|$ realize a projection onto the superposition of states $|\Phi _{1,2}\rangle$, which generally are not orthogonal to each other obeying the condition

41 $$\begin{aligned} \left\langle \Phi _1\Big |\Phi _2\right\rangle = \eta . \end{aligned}$$

Simultaneously, we require the states in Eqs. (39) and (40) to meet the normalization condition

42 $$\begin{aligned} \left\langle \pi _i\Big |\pi _j\right\rangle = \delta _{ij},\quad i,j=0,1. \end{aligned}$$

Now, we are able to determine the coefficients $c_{1,2}$, which fulfill Eqs. (41) and (42) and look like

43 $$\begin{aligned} c_{1,2}=\sqrt{\frac{1\pm \sqrt{1-\eta ^2}}{2(1-\eta ^2)}}. \end{aligned}$$

In Eqs. (41) and (43), the parameter $\eta$ defines the distinguishability of states $|\Phi _{1,2}\rangle$. The case of $\eta =1$ corresponds to completely indistinguishable states $|\Phi _{1,2}\rangle$. In this case one can assume that $|\Phi _{1}\rangle$ and $|\Phi _{2}\rangle$ represent the same state.

On the other hand, the case of $\eta =0$ ($c_1=1$ and $c_2=0$) characterizes completely orthogonal states $|\Phi _{1,2}\rangle$; that becomes possible if $|\Phi _{1,2}\rangle$ approach two-mode Fock states. In other words, this is a limit of the *N*00*N*-state, for which the coupled solitons are examined.

For $\eta \ne 1$ it is instructive to represent soliton wave functions shown in Eqs. (13) and (14) in the form of

44 $$\begin{aligned} \Psi _1= & {} \frac{{\sqrt{uN}}}{4}(1-z){{\,\text{ {sech}}\,}}\left[ \left( 1-z\right) \left( \frac{uN}{4}x-\Delta \right) \right] e^{-i\frac{\theta }{2}}, \end{aligned}$$

45 $$\begin{aligned} \Psi _2= & {} \frac{{\sqrt{uN}}}{4}(1+z){{\,\text{ {sech}}\,}}\left[ \left( 1+z\right) \left( \frac{uN}{4}x+\Delta \right) \right] e^{i\frac{\theta }{2}}. \end{aligned}$$

To be more specific, we examine here two soliton interaction with relative phase $\Theta =0$ and intersoliton distances above critical values. From Eq. (6) we obtain

46 $$\begin{aligned} \left| \Phi _1\right\rangle= & {} \frac{1}{\sqrt{N!}}\left[ \int _{-\infty }^\infty \left( \Psi _1^{(+)}{\hat{a}}_1^\dag + \Psi _2^{(+)}{\hat{a}}_2^\dag \right) dx \right] ^N\left| 0\right\rangle ,\end{aligned}$$

47 $$\begin{aligned} \left| \Phi _2\right\rangle= & {} \frac{1}{\sqrt{N!}}\left[ \int _{-\infty }^\infty \left( \Psi _1^{(-)}{\hat{a}}_1^\dag + \Psi _2^{(-)}{\hat{a}}_2^\dag \right) dx \right] ^N\left| 0\right\rangle , \end{aligned}$$

for two “halves” of the SC-state, where

48 $$\begin{aligned} \Psi _1^{(\pm )}= & {} \frac{\sqrt{uN}}{4}(1-z_{\pm }){{\,\text{ {sech}}\,}}\left[ \left( 1-z_{\pm }\right) \left( \frac{uN}{4}x - \Delta \right) \right] , \end{aligned}$$

49 $$\begin{aligned} \Psi _2^{({\pm })}= & {} \frac{\sqrt{uN}}{4}(1+z_{\pm }){{\,\text{ {sech}}\,}}\left[ \left( 1+z_{\pm }\right) \left( \frac{uN}{4}x + \Delta \right) \right] . \end{aligned}$$

In Eqs. (48) and (49), $z_{+}$ and $z_{-}$ are two SS solutions corresponding to the upper and lower branches in Fig. 3, respectively. $e^{-iN(\theta /2 + \beta _1\tau )}$. In particular, for $\Omega \approx 0$, we have $z_{\pm }\rightarrow \pm z_0$.

The scalar product for state given in Eqs. (46) and (47) is

50 $$\begin{aligned} \eta = \left[ \int _{-\infty }^\infty \left( \Psi _1^{(+)}\Psi _1^{(-)} + \Psi _2^{(+)}\Psi _2^{(-)}\right) dx\right] ^N\equiv \varepsilon ^N, \end{aligned}$$

where $\varepsilon$ characterizes solitons wave functions overlapping. Assuming non-zero and positive $\Omega$ for $\varepsilon$, one can obtain

51 $$\begin{aligned} \varepsilon= & {} \frac{1}{2}\Bigg (\left( 1-\frac{z_++z_-}{2}\right) ^{{-1}} \left( 1-z_+\right) \left( 1-z_-\right) \left( 1-0.21\left[ \frac{z_+-z_-}{2-(z_+ + z_-)}\right] ^2\right) \nonumber \\&+\left( 1+\frac{z_++z_-}{2}\right) ^{{-1}}\left( 1+z_+\right) \left( 1+z_-\right) \left( 1-0.21\left[ \frac{z_+-z_-}{2+(z_++z_-)}\right] ^2\right) \Bigg ). \end{aligned}$$

**Figure 7:**
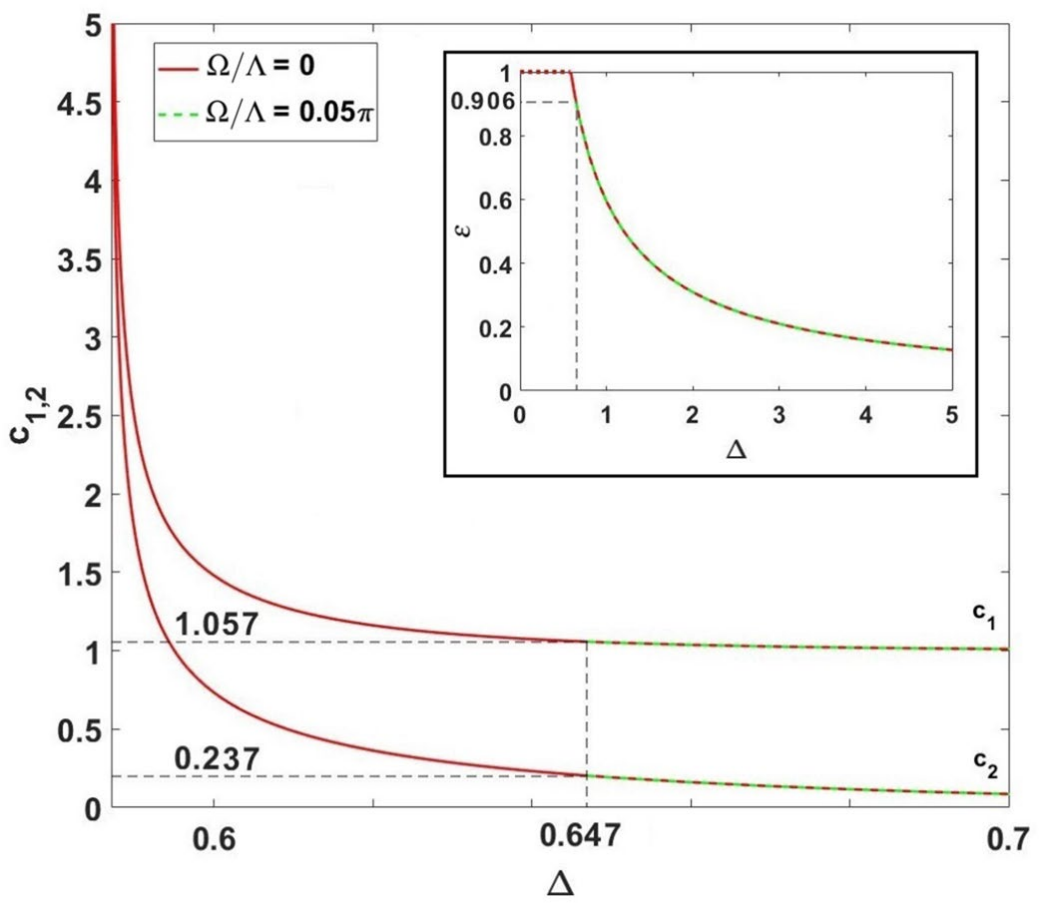
Coefficients $c_{1,2}$ versus the normalized inter-soliton distance $\Delta$ for $N=10$ and different $\Omega {/\Lambda }$. The inset demonstrates the behavior of $\varepsilon$ for different $\Delta$. $\Delta _c^{ub}=0.647$ corresponds to the intersoliton distance of the upper-branch SS appearence at $\Omega /\Lambda = 0.05\pi$, which is crucial for the SC-state formation.

In Fig. 7, we establish the principal features of coefficients shown in Eq. (43) and parameter $\varepsilon$, see the inset in Fig. 7, as functions of $\Delta$. The value of $\Omega$ plays a significant role in the distinguishability problem for states $|\Phi _{1}\rangle$ and $|\Phi _{2}\rangle$. In particular, for $\Omega =0$ at the bifurcation point $\Delta =\Delta _c=0.5867$, we have $\varepsilon =1$ that implies indistinguishable states $|\Phi _{1}\rangle$ and $|\Phi _{2}\rangle$, see the red curve in the inset of Fig. 7. In this limit, the coefficients $c_{1,2}\rightarrow \infty$.

However, even for the small (positive) $\Omega$, it follows from Eq. (51) that $\varepsilon \ne 1$ for any $\Delta >\Delta _c$, and states $|\Phi _{1}\rangle$, $|\Phi _{2}\rangle$ are always distinguishable. In particular, it follows from zero-phase solution given in Eq. (35) that $z_{\pm } = \pm \left( 1.2 - \frac{18\Delta _-}{{\omega _{ST}^2}}\right) \sqrt{\Delta _-} - \frac{\Omega }{{\Lambda }}\frac{f(\Delta _-)}{{\omega _{ST}^2}}$ and $|z_{+}|\ne |z_{-}|$.

This is displayed by the green curves in Fig. 7. The SS solutions possess $c_1=1.057$, $c_2=0.203$ for $c_{1,2}$ that correspond to $\Delta _c^{ub} \simeq 0.647$, $\varepsilon \approx 0.9056$ for $\Omega {/\Lambda }=0.05\pi$.

From Fig. 7, it is evident that coefficients $c_{1,2}$ rapidly approach (due to the factor *N*) levels $c_{1}=1$, $c_{2}=0$ (completely distinguishable macroscopic SC soliton states), when $\Delta$ increases. In this limit, as seen from Fig. 3, $z_{\pm }$ approaches $\pm z_0$, and from Eq. (51) we obtain

52 $$\begin{aligned} \varepsilon \approx (1-z_0^2)(1-0.21z_0^2). \end{aligned}$$

Practically, in this limit the red and green curves coincide in Fig. 7.

Remarkably, the case of $\eta =0$ characterizes completely orthogonal states $|\Phi _{1,2}\rangle$ in (41) and (50); that becomes possible if $|\Phi _{1,2}\rangle$ approach two-mode Fock states. In other words, this is a limit of the *N*00*N*-state, for which we examine the coupled solitons. In this limit, the relative soliton phase approaches (23).

### Parameter estimation with macroscopic qubit states

We can exploit states shown in Eqs. (46) and (47) in metrological measurement for arbitrary phase $\phi _N$ estimation. In general, suppose that two-soliton quantum system is prepared in state $|\psi \rangle$, which carries information about some parameter $\Gamma$ that we would like to estimate. In this work we are interested in fundamental bound for a positive operator valued measurement (POVM) and consider pure states of the quantum system.

The precision of the estimation of some parameter $\Gamma$ is described by the error propagation formula^27^ given as

53 $$\begin{aligned} \sigma _\Gamma = \frac{\sqrt{\left\langle \psi |(\Delta {\hat{\Pi }} )^2|\psi \right\rangle }}{\left| \frac{\partial \left\langle \psi | {\hat{\Pi }}|\psi \right\rangle }{\partial \Gamma }\right| }, \end{aligned}$$

where within the quantum metrology approach $\left\langle \psi |(\Delta {\hat{\Pi }})^2|\psi \right\rangle = {\left\langle \psi |{\hat{\Pi }}^2 |\psi \right\rangle - \left\langle \psi |{\hat{\Pi }}|\psi \right\rangle }^2$ represents the variance of fluctuations of some operator ${\hat{\Pi }}$ that corresponds to the measurement procedure performed with the (pure) state $|\psi \rangle$. Typically, such procedures are based on appropriate interferometric schemes and use quantum superpositions initially prepared and then explored for measurement and estimation of parameter $\Gamma$, cf.^76^. In other words, the measurement procedure, that we consider here, includes three important steps involving two-soliton state preparation, subsequent phase $\phi _N$ accumulation and measurement (estimation), cf.^9^. Practically, two-soliton state preparation involves a splitting procedure, which corresponds to the first beam splitter action in traditional Mach–Zehnder interferometer, see e.g.^10^. At this stage we suppose $\Omega$ vanishing.

Then, we assume that the output state $|\psi \rangle$ of quantum system that we use for measurement and parameter (phase) estimation is represented as

54 $$\begin{aligned} |\psi \rangle =\frac{1}{\sqrt{2}}(|\pi _0\rangle {-}e^{i\phi _N}|\pi _1\rangle ), \end{aligned}$$

where $\phi _N$ is a relative (estimated) phase between states $|\pi _0\rangle$ and $|\pi _1\rangle$, defined as

55 $$\begin{aligned} \phi _N=N\Theta _{\Omega }+N\varphi . \end{aligned}$$

In (55) $\Theta _{\Omega }$ is the phase occurring between the solitons within the time $\tau _{\Omega }$ for non-vanishing $\Omega$; $\varphi =\omega \tau _{\omega }\equiv \Gamma$ is an additional phase accumulated during time $\tau _{\omega }$. Thus, we suppose that the measurement and parameter-estimation procedure generally includes two stages characterized by total phase $\phi _N$.

At first, let us examine the problem of estimation some arbitrary phase $\varphi$, which may be created after two-soliton state formation. The role of soliton phase difference $\Theta _{\Omega }$ is negligible here, if we consider soliton interaction regimes with vanishing $\Omega$ (or very short time interval $\tau _{\Omega }$), proposing $\Theta =0$, or $\Theta =\pi$. In this limit we exploit soliton SC-state to estimate phase parameter $\phi _N\simeq N\varphi$. We assume in (54) that phase $\phi _N\simeq N\Gamma$ contains all the information about measured parameter $\Gamma$ and linearly depends on particle number *N*. Notice, this assumption is valid only in the linear metrology approach framework, cf.^30^.

Then, we define a complete set of operators ${\hat{\Sigma }}_j$, $j=1,2,3$ (cf.^60^)

56 $$\begin{aligned} {\hat{\Sigma }}_0= & {} \left| \pi _1\right\rangle \left\langle \pi _1\right| + \left| \pi _0\right\rangle \left\langle \pi _0\right| , \end{aligned}$$

57 $$\begin{aligned} {\hat{\Sigma }}_1= & {} \left| \pi _1\right\rangle \left\langle \pi _1\right| - \left| \pi _0\right\rangle \left\langle \pi _0\right| , \end{aligned}$$

58 $$\begin{aligned} {\hat{\Sigma }}_2= & {} \left| \pi _0\right\rangle \left\langle \pi _1\right| + \left| \pi _1\right\rangle \left\langle \pi _0\right| , \end{aligned}$$

59 $$\begin{aligned} {\hat{\Sigma }}_3= & {} i( \left| \pi _0\right\rangle \left\langle \pi _1\right| - \left| \pi _1\right\rangle \left\langle \pi _0\right| ), \end{aligned}$$

which obey the SU(2) algebra commutation relation.

The meaning of sigma-operators is evident from their definitions given in Eqs. (56)–(59). Physically, operators (56)–(59) are similar to the Stokes parameters, which characterize polarization qubit (two-mode) state, cf.^77^ . Due to the properties shown in Eq. (42), the states $|\pi _{i}\rangle$ are suitable candidates for the macroscopic qubit states, which we can define by mapping $|\pi _{0}\rangle \rightarrow |{\mathbf {0}}\rangle$ and $|\pi _{1}\rangle \rightarrow |{\mathbf {1}}\rangle$, respectively^53,78^. In this form we can use them for POVM measurements defined with operators^79^

60 $$\begin{aligned} E_1\equiv & {} \frac{\sqrt{2}}{1+\sqrt{2}}|{\mathbf {1}}\rangle \left\langle {\mathbf {1}} \right| =\frac{1}{\sqrt{2}(1+\sqrt{2})}({\hat{\Sigma }}_0+{\hat{\Sigma }}_1), \end{aligned}$$

61 $$\begin{aligned} E_2\equiv & {} \frac{1}{\sqrt{2}(1+\sqrt{2})}(|{\mathbf {0}}\rangle -|{\mathbf {1}}\rangle )(\langle {\mathbf {0}}| + \langle {\mathbf {1}}|) =-\frac{1}{\sqrt{2}(1+\sqrt{2})}({\hat{\Sigma }}_1+i{\hat{\Sigma }}_3), \end{aligned}$$

62 $$\begin{aligned} E_3\equiv & {} I-E_1-E_2. \end{aligned}$$

Importantly, current quantum technologies permit POVM tomography^54^.In particular, POVM tomography involves reconstruction of the Stokes vector for the polarization qubit and requires four measurements at least, cf.^80^. In Ref.^81^ we suggested a special multiport interferometer for simultaneous measurement of all the Stokes parameters, which are relevant to macroscopic two-mode quantum state characterization. The proposed interferometer consists of a set of beam splitters and simple phase-shift device, which may be implemented in the atomic optics domain by relevant procedures performed with two-mode (spinor) atomic condensates, cf.^9,10^.

Average values of sigma-operators in Eqs. (56)–(59) can be obtained with the help of Eqs. (42) and (54), resulting in

63 $$\begin{aligned} \left\langle {\hat{\Sigma }}_1\right\rangle= & {} 0, \end{aligned}$$

64 $$\begin{aligned} \left\langle {\hat{\Sigma }}_2\right\rangle= & {} \cos [\phi _N],\end{aligned}$$

65 $$\begin{aligned} \left\langle {\hat{\Sigma }}_3\right\rangle= & {} \sin [\phi _N]. \end{aligned}$$

From Eqs. (63)–(65) it follows that only $\left\langle {\hat{\Sigma }}_{2,3}\right\rangle$ contain the information about the desired phase $\phi _N$.

To estimate the sensitivity of phase measurement, we can assume here $\phi _N=N\Gamma$ and use Eq. (53) with the measured operator ${\hat{\Pi }}\equiv \hat{\Sigma _2}$. Taking into account $\left\langle {\hat{\Sigma }}_2^2\right\rangle = 1$ for the variance of fluctuations $\left\langle (\Delta {\hat{\Sigma }}_2)^2\right\rangle$, we obtain

66 $$\begin{aligned} \left\langle (\Delta {\hat{\Sigma }}_2)^2\right\rangle = \sin ^2[N\Gamma ]. \end{aligned}$$

Finally, from Eqs. (53) and (66) for the phase error propagation we get

47 $$\begin{aligned} \sigma _\Gamma = \frac{1}{N}, \end{aligned}$$

that clearly corresponds to the HL of arbitrary (linearly *N*-dependent) phase estimation and explores the sigma-operator measurement procedure.

### Nonlinear metrology approach for frequency measurement, $\Gamma \equiv \Omega$

Now, we represent another particularly important case which relates to angular frequency $\Omega$ measurement that characterizes energy spacing between the ground and first excited macroscopic states. In particular, we suppose that all the information about $\Omega$ is embodied in phase $\phi _N=N\Theta _{\Omega }$; the measurement and estimation procedure is realized immediately after period $\tau _{\Omega }$. In other words, here we ignore linear (in respect of particle number *N*, frequency $\omega$ and time duration $\tau _{\omega }$) phase shift $\varphi$, cf. (55).

The SS solutions given in (22) and (23), which correspond to the maximal population imbalance, $z^2=1$, allow us to prepare the maximally path-entangled superposition state, a.k.a. *N*00*N*-state. As seen from Eq. (23), the solution with $z=1$ exists when $-2(\pi -1)\le \Omega /\Lambda \le 2(\pi +1)$. Similarly, the domain of solution $z=-1$ is $-2(\pi +1)\le \Omega /\Lambda \le 2(\pi -1)$. To achieve the superposition *N*00*N*-state formation, we require both solutions to exist simultaneously. This restricts the domain of $\Omega$ as $-2(\pi -1)\le \Omega /\Lambda \le 2(\pi -1)$.

Substituting $z =\pm 1$ into Eqs. (44) and (45) we obtain

68 $$\begin{aligned} \Psi _1= & {} \frac{\sqrt{uN}}{2}{{\,\text{ {sech}}\,}}\left[ \left( \frac{uN}{2}x-2\Delta \right) \right] e^{-i\frac{\theta }{2}}, \end{aligned}$$

69 $$\begin{aligned} \Psi _2= & {} \frac{\sqrt{uN}}{2}{{\,\text{ {sech}}\,}}\left[ \left( \frac{uN}{2}x+2\Delta \right) \right] e^{i\frac{\theta }{2}}, \end{aligned}$$

which are relevant to the *N*00*N*-state’s two “halves” defined as

70 $$\begin{aligned} \left| N0\right\rangle= & {} \frac{1}{\sqrt{N!}}\left[ \int _{-\infty }^\infty \Psi _1{\hat{a}}_1^\dag dx\right] ^N\left| 0\right\rangle, \end{aligned}$$

71 $$\begin{aligned} \left| 0N\right\rangle= & {} \frac{1}{\sqrt{N!}}\left[ \int _{-\infty }^\infty \Psi _2{\hat{a}}_2^\dag dx\right] ^N\left| 0\right\rangle . \end{aligned}$$

Considering the superposition of states shown in Eqs. (70) and (71) and omitting unimportant common phase $e^{iN\left( 0.5\theta ^{(-)}-\beta _1t\right) }$, from (54) we arrive at

72 $$\begin{aligned} {|\psi \rangle \equiv }\left| N00N\right\rangle = \frac{1}{\sqrt{2}} \left( \left| N0\right\rangle + e^{iN{\Theta _{\Omega }}}\left| 0N\right\rangle \right) , \end{aligned}$$

that represents the *N*00*N*-state of coupled BEC solitons for our problem. Here,

73 $$\begin{aligned} {\Theta _{\Omega }} = \frac{\Theta ^{(+)} + \Theta ^{(-)}}{2} = \frac{1}{2}\left( \arccos \left[ \frac{2\Lambda - \Omega }{2\pi \Lambda }\right] + \arccos \left[ \frac{2\Lambda + \Omega }{2\pi \Lambda }\right] \right) , \end{aligned}$$

is the phase shift that contains the $\Omega$-parameter required for estimation.

Remarkably, we deal here with the nonlinear metrology approach since two-soliton phase $\Theta _{\Omega }$ nonlinearly depends on $\Omega$ and parameter $\Lambda$ (particle number *N*), cf.^31,32,47^.

To study the ultimate achievable precision of such a measurement with state (72), we consider the quantum Cramer–Rao bound^9^

74 $$\begin{aligned} \sigma _\Omega = \frac{1}{\sqrt{\nu F_Q}}, \end{aligned}$$

where $\nu$ is the number of subsequent measurements (for the sake of simplicity we take $\nu =1$) and $F_Q$ is the quantum Fisher information. The latter is defined for the pure state $\left| \psi \right\rangle$ of the system as

75 $$\begin{aligned} F_Q = 4\left[ \left\langle \psi '_\Omega |\psi '_\Omega \right\rangle - \left| \left\langle \psi |\psi '_\Omega \right\rangle \right| ^2\right] , \end{aligned}$$

where $\left| \psi '_\Omega \right\rangle \equiv \partial \left| \psi \right\rangle /\partial \Omega$. Substituting (72) with $|\psi \rangle \equiv |N00N\rangle$ into (75) and then into (74) we get

76 $$\begin{aligned} \sigma _\Omega = \frac{1}{N}\left| \frac{\partial {\Theta _{\Omega }}}{\partial \Omega }\right| ^{-1}. \end{aligned}$$

Notice, Eq. (76) directly results from (53) with (72) and (73).

Comparing Eq. (72) with Eq. (54), we can conclude that the *N*00*N*-state “halves” $\left| N0\right\rangle$ and $\left| 0N\right\rangle$ in Eq. (72) may be associated with states $|\pi _0\rangle$ and $|\pi _1\rangle$, respectively. To estimate the sensitivity of the $\Omega$-measurement, we use Eq. (76) with measured operator ${\hat{\Pi }}\equiv {\hat{\Sigma }}$ defined as

77 $$\begin{aligned} {\hat{\Sigma }} = \left| N0\right\rangle \left\langle 0N\right| + \left| 0N\right\rangle \left\langle N0\right|. \end{aligned}$$

Since states shown in Eq. (77) are orthogonal, the mean value of Eq. (77) is

78 $$\begin{aligned} \left\langle {\hat{\Sigma }}\right\rangle = \cos [N\Theta _{{\Omega }}]. \end{aligned}$$

**Figure 8:**
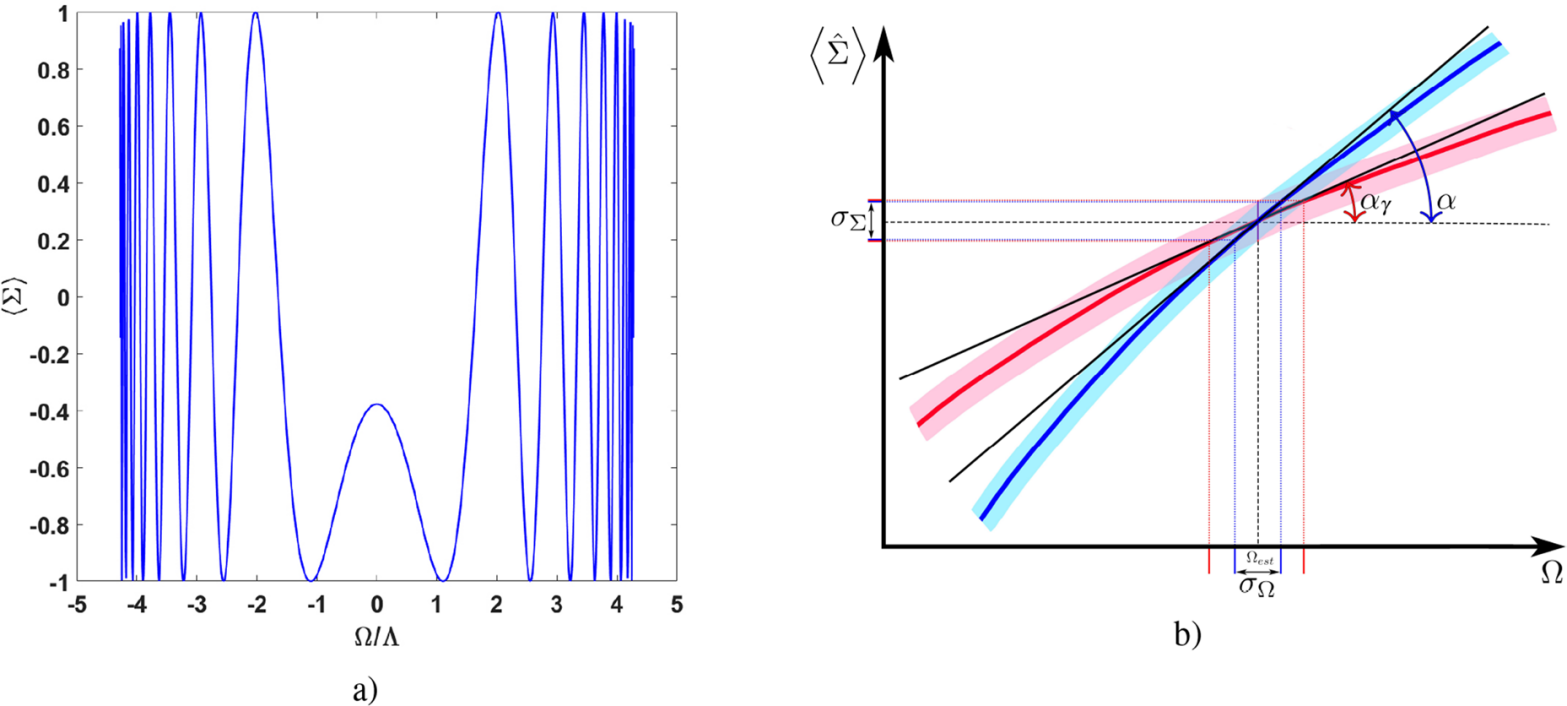
(**a**) Mean value $\left\langle {\hat{\Sigma }}\right\rangle$ vs. normalized angular frequency $\Omega /\Lambda$ for (**a**) the whole interval of $\Omega$, and (**b**) in the vicinity of estimated value $\Omega _{est}$, respectively. The particle number is $N=200$. In (**b**) the blue and red curves characterize some fragment of the interference pattern without and with incoherent (extra) noises, respectively. The solid black lines denote tangents to these curves. Relevant angles $\alpha$ ($\tan [\alpha ]={\left| \frac{\partial \ \left\langle {\hat{\Sigma }}\right\rangle }{\partial \Omega }\right| }$) and $\alpha _\gamma$ ($\tan [\alpha _\gamma ]=\left| \frac{\partial \ \left\langle {\hat{\Sigma }}\right\rangle _\gamma }{\partial \Omega }\right|$) describe the tangent slopes at the points corresponding to the estimated parameter $\Omega _{est}$ without and with weak losses $\gamma$, respectively; $\left\langle {\hat{\Sigma }}\right\rangle _\gamma$ is the mean value of measured parameter $\Sigma$ in the presence of losses. For the blue curve some uncertainty $\sigma _{\Sigma }\equiv \sqrt{\left\langle (\Delta {\hat{\Sigma }})^2\right\rangle }$ is shown evoked by frequency $\Omega$ measurement and estimation performed with error $\sigma _{\Omega }$. The shadow regions exhibit relevant uncertainties in $\Sigma$ as functions of $\Omega$. See more details in the text.

Figure 8a demonstrates $\left\langle {\hat{\Sigma }}\right\rangle$ as a function of $\Omega /\Lambda$. Notice, the interference pattern in Fig. 8a exhibits an essentially nonlinear behavior for measured $\left\langle {\hat{\Sigma }}\right\rangle$.

The variance of fluctuations $\left\langle (\Delta {\hat{\Sigma }})^2\right\rangle$ for the measured sigma-operator reads as

79 $$\begin{aligned} \left\langle (\Delta {\hat{\Sigma }})^2\right\rangle = \sin ^2[N\Theta _{{\Omega }}]. \end{aligned}$$

Now, by using Eqs. (73) and (76) we can easily find the propagation error for the $\Omega$-estimation as

80 $$\begin{aligned} \sigma _\Omega = \frac{2\Lambda }{N}\left| \frac{\sqrt{4\pi ^2 -(2+\Omega /\Lambda )^2}\sqrt{4\pi ^2-(2-\Omega /\Lambda )^2}}{\sqrt{4\pi ^2-(2+\Omega /\Lambda )^2} - \sqrt{4\pi ^2-(2-\Omega /\Lambda )^2}}\right| . \end{aligned}$$

Then, we choose the optimal estimation area for $\Omega$, with the best sensitivity reached, in the vicinity of the domain border at $\Omega /\Lambda \rightarrow 2(\pi -1)$. In this limit, Eq. (80) approaches

81 $$\begin{aligned} \sigma _\Omega \simeq \frac{10\Lambda }{N}\frac{1.17\sqrt{2(\pi -1) - \Omega /\Lambda }}{1.65 - \sqrt{2(\pi -1) - \Omega /\Lambda }}. \end{aligned}$$

Equations (80) and (81) demonstrate one of the important results of this paper: for a given $\Lambda$, that characterizes atomic condensate peculiarities, Eq. (81) demonstrates Heisenberg scaling for frequency measurement sensitivity.

At the same, time (80) and (81) exhibit some specific peculiarities in two limiting cases, which are inherent to the highly nonlinear interference pattern represented in Fig. 8a.

First, (80) is non-applicable ($\sigma _\Omega \rightarrow \infty$ ) for $\Omega =0$ since ${\left| \frac{\partial \ \left\langle {\hat{\Sigma }}\right\rangle }{\partial \Omega }\right| =0}$.

Second, $$\sigma _\Omega \rightarrow 0$$ if $$\Omega /\Lambda \rightarrow 2(\pi -1)$$.

Qualitatively this situation is shown in Fig. 8b. Geometrically $\tan [\alpha ]={\left| \frac{\partial \ \left\langle {\hat{\Sigma }}\right\rangle }{\partial \Omega }\right| }$ determines the slope of the tangent to the curve characterizing the interference pattern, cf.^27^. The blue curve in Fig. 8b corresponds to the ideal interference pattern shown in Fig. 8a. Obviously, the tangent is parallel to the abscissa axis in $\Omega =0$ point. At the border of the pattern the tangent tends to be perpendicular to the $\Omega$ axis and $\sigma _\Omega \rightarrow 0$. Physically, such a behavior is not surprising and corresponds to the essentially nonlinear (in respect of $\Omega$ ) metrology limit that establishes the interference pattern in Fig. 8a. In this case one can obtain the SH scaling for the phase parameter measurement and estimation, cf.^30,32–34^.

### Experimental feasibility of quantum metrology with condensate bright solitons

Let us briefly discuss the feasibility of experimental observation of the proposed high-precision measurements with mesoscopic superposition states in the presence of losses for the quantum soliton system in Fig. 1. A purely quantum theory (beyond the Hartree approach) of coupled solitons established in Fig. 1 represents a non-trivial task due to the essentially nonlinear character of particle tunneling between the solitons. However, the results for quantum solitons obtained in^48^ allow to present here some simple arguments on the feasibility of quantum-enhanced metrology effects observation with coupled solitons discussed above.

First, we examine the influence of particle losses on *N*00*N*-state (72). The losses that occur between the two-soliton quantum state preparation and the measurement are similar to the action of some fictitious beam splitters, which introduce additional noises^36,48^. As shown in^48^, the resulting quantum state of coupled (in the transverse plane) solitons may be characterized by the superposition of the Fock states with highly populated *N*00*N*-components. In the presence of few (even one) particle losses such a state experiences a partial collapse with the formation of the highly asymmetric *N*00*N*-like state. We represent here such a state as

82 $$\begin{aligned} \left| N00N\right\rangle _{\gamma } = \frac{1}{\sqrt{1+\gamma ^2}}\left( \gamma \left| N0\right\rangle + e^{iN\Theta _{\Omega }}\left| 0N\right\rangle \right) , \end{aligned}$$

where $\gamma$ is a vanishing parameter characterizing the *N*00*N*-state decay in the presence of losses. $\gamma$ may be estimated as $\gamma \simeq 0.25 N^{-1/2}$ if one particle is lost from the coupled solitons, see for details^48^. Now, instead of (78) we obtain

83 $$\begin{aligned} \left\langle {\hat{\Sigma }}\right\rangle \equiv \left\langle {\hat{\Sigma }} \right\rangle _\gamma = \frac{2\gamma }{1+\gamma ^2}\cos [N\Theta _{{\Omega }}]. \end{aligned}$$

Equation (83) manifests a vanishing interference pattern in the limit of $\gamma \rightarrow 0$. Equations (78) and (83) allow to establish relations between angles $\alpha$ and $\alpha _\gamma$:

84 $$\begin{aligned} \tan [\alpha _\gamma ] =\frac{2\gamma }{1+\gamma ^2}\tan [\alpha ]\simeq \frac{1}{2\sqrt{N}}\tan [\alpha ]. \end{aligned}$$

The last relation in (84) is valid for a single particle loss.

Equation (84) possesses a simple geometric interpretation: the particle losses reduce the slope of the tangent to the curve more than $\sqrt{N}$ times. Moreover, from definition Eqs. (53) and (82) it is possible to show that the propagation error for the $\Omega$-estimation in the presence of losses that we define as $\sigma _{\Omega ,\gamma }$ also increases in $2\sqrt{N}$ times in comparison with $\sigma _{\Omega }$ established in (80). The red curve in Fig. 8b qualitatively demonstrates how losses case the decrease of the $\alpha$ angle and increase of $\sigma _\Omega$.

Thus, losses lead to the decoherence of an interference pattern, cf.^82^, and, as a result, the slope of the curve modifies. Hence, in the real-world $\Omega$ measurement, for essential amount of losses the accuracy $\sigma _{\Omega ,\gamma }\propto 1/\sqrt{N}$ corresponds to the standard quantum limit of frequency estimation.

It is worth to notice that qubit states  (39) and (40) based on SC-state solutions for $|\Phi _1\rangle$ and $|\Phi _2\rangle$ “halves” should be more robust to small particle losses since each “halve” posses a binomial distribution of mesoscopic (or macroscopic) number of particles, see (46) and (47).

Now let us discuss which type of losses are actual for two-soliton states and how we can avoid them obtaining quantum-enhanced metrology discussed above.

Previously, in Ref.^48^, and then in Ref.^84^ we examined this problem for quantum solitons possessing simple Josephson coupling in another, transverse, configuration of soliton interaction, which is reminiscent to commonly considered weakly-coupled condensates possessing Gaussian wave functions, cf.^12–14,16^. From the experimental point of view, recent BEC soliton experiments with lithium condensates demonstrated that one- and three-body losses may be recognized as major detrimental effects for quantum solitons, cf.^48,85,86^.

In particular, we examine here time scale $\tau _d$, in which an one-particle-loss event takes place in average; $\tau _d$ may be calculated as (cf.^85,86^)

85 $$\begin{aligned} \tau _d = \left( \frac{N}{\tau _1} + \frac{N^5}{3\tau _3}\right) ^{-1}, \end{aligned}$$

where $\tau _1\equiv 1/K_1$ and $\tau _3$ are characteristic times for one- and three-body losses, respectively. The temporal parameters introduced in (85) are dimensionless (we normalized time variable on characteristic time scale $1/\omega _{\perp }$, as it is established in (1)). Notice, apart from our approach represented in Ref.^48^, authors in Refs.^85,86^ take into account the density heterogeneity within soliton spatial distribution, which implies the fifth order power in respect of particle number *N* in Eq. (85) for three-body recombination losses. We can represent $\tau _3$ in terms of dimensionless nonlinear strength *u* as

86 $$\begin{aligned} \tau _3 = \frac{90\pi ^2}{u^2K_3}\equiv \frac{5.625\pi ^2 N^2}{\Lambda K_3}, \end{aligned}$$

where $K_3$ is a constant (normalized on $\omega _\perp a_{\perp }^6$) responsible for three-body recombination losses.

Another important characteristic time is

87 $$\begin{aligned} \tau _{sol} = \frac{1}{u^2N^2}\equiv \frac{1}{16\Lambda }, \end{aligned}$$

which results from the energy-time uncertainty relation. We consider particle losses as adiabatic processes occurring slowly in comparison with $\tau _{sol}$, cf.^15,85,86^.

Thus, we can impose that the changes in particle number *N* (loss events) should take place on time scales sufficiently longer than characteristic time scales $\tau _{sol}, \tau _{\Omega }, \tau _{\omega }$. Strictly speaking, we require

88 $$\begin{aligned} \tau _{sol}< \tau _{\Omega }, \tau _{\omega } < \tau _{d}, \end{aligned}$$

as a necessary condition for observation of the proposed measurement and estimation approach with solitons.

For numerical estimations we use here experimentally accessible parameters of bright solitons observed with lithium BEC^42^. A harmonic magneto-optical potential was exploited to trap the BEC of ${}^7$Li atoms with characteristic frequency $\omega _\perp = 2\pi \times 710$ Hz, providing characteristic spatial scale $a_\perp =1.4\times 10^{-6}$ m. The condensate soliton was formed at s-wave scattering length $a_{sc}=-0.21\times 10^{-9}$ m manipulated via the Feshbach resonance technique.

Coefficients $K_1$ and $K_3$ for one- and three-body losses may be estimated (in physical units) as $K_1=0.05\,\hbox {s}{}^{-1}$ and $K_3=6\times 10^{-42}\,\hbox {m}^6\hbox {s}^{-1}$, respectively, cf.^86^.

Finally, for mesoscopic particle number $N\simeq 1000$ from (85) we obtain estimation characteristic time scales as $\tau _d = 87.4$, $\tau _{sol} = 0.28$ ($\Lambda \approx 0.22$), which imply $\tau _d \simeq 19.6$ ms and $\tau _{sol} = 63$ $\mu$s given in physical units, respectively. It is worth noticing that three-body losses are quite small in this limit and may be neglected, cf.^48^. Our estimations show that the last term in (85) relevant to three-body losses becomes compatible with the first one for particle number $N\simeq 3000$. Obviously, three-body losses dominate with increasing particle number *N*, cf.^86^. However, bright solitons occurring in the condensates with a negative scattering length possess a wave-function collapse for macroscopically large *N*^42^. Roughly speaking, condensate bright solitons collapse if the number of atoms exceeds the critical value, $N_c=0.67 a_{\perp }/|a_{sc}|$^39^, which implies $uN_c\approx 4.2$ (in Ref.^42^ authors demonstrated that $N_c$ is relevant to the number of atoms $5.2\times 10^3$). In Refs.^48,84^ we proposed coupled bright solitons for quantum metrology purposes containing few hundreds of condensate particles.

Thus, our approach for the measurement and parameter estimation procedure requires time scales shorter than $\tau _d$ and a moderate (mesoscopic) number of condensate particles, see (88). Notice, that in Ref.^42^ the observation time was less than 10 ms. Quantum properties of solitons applied to metrology will become possible with further improvements (in respect of particle number) of current experiments with BEC solitons^43^ and cf.^84^.

In the real-world experiment, on-chip Mach–Zehnder interferometer technology for atomic condensates may be useful for the frequency parameter $\Omega$ estimation described above, cf.^10^. In particular, an accumulated (additional) phase $\varphi$ can also help select a measurement window in respect to parameter $\Omega$ for the interference pattern given in Fig. 8a. The magnetic field can be implemented for $\Omega$ tuning and manipulation. At the same time, magnetic field may be used to tune and enhance atom-atom scattering length^11^. Furthermore, the accuracy at Heisenberg scaling and beyond for parameter (phase) estimation alternatively may be obtained by the parity measurement procedure instead of ${\hat{\Sigma }}$-operator exploring, see^47,60^ and, especially, Ref.^83^ for recent progress achieved with the parity-based detection technique for atomic quantum states. Thus, we expect the on-chip Mach–Zehnder interferometer containing soliton Josephson junctions to be in focus of experimental research in the near future.

## Conclusion

In summary, we have considered the problem of two-soliton formation for 1D BECs trapped effectively in a double-well potential. The analytical solutions of these soliton Josephson junctions and corresponding phase portraits exhibit the occurrence of novel macroscopic quantum selft-trapping (MQST) phases in contrast to the condensates with only Gaussian wave functions. With these soliton states, we have also explored the formation of the Schrödinger-cat (SC) state in the framework of the Hartree approximation. In particular, we have analyzed the distinguishability problem for binary (non-orthogonal) macroscopic states. Compared to the known results^47^, finite frequency spacing $\Omega$ leads to distinguishable macroscopic states for condensate solitons. This circumstance may be important for the experimental design of the SC-states.

The important part of this work is devoted to the applicability of predicted states for quantum metrology. In the framework of the linear metrology approach, by utilizing the macroscopic qubits problem with the interacting BEC solitons, one can apply the sigma-operators to elucidate the measurement and subsequent estimation of an arbitrary phase, that linearly depends on the particle number, up to the HL. Notably, the sigma-operators relate to the POVM detection tomography, which is similar to the Stokes parameters measurement within the qubit state reconstruction procedure. On the other hand, the phase estimation procedure for the phase-dependent sigma-operator can be realized by means of the parity measurement technique that produces the same accuracy for phase estimation. We have shown that in the limit of soliton state solution with the population imbalance $|z|=\pm 1$ the coupled soliton system admits the maximally path-entangled *N*00*N*-state formation.

In the framework of the nonlinear metrology approach, we have also demonstrated the possibility to estimate frequency $\Omega$ at the HL and beyond by soliton phase difference estimation. The SH scaling for frequency estimation becomes possible due to the nonlinear interference pattern, which occurs for the relative soliton phase.

The numerical estimation for characteristic time scales of one- and three-body losses which are based on the existing experimental results with condensate bright solitons, demonstrates the feasibility of the experimental realization of the proposed quantum metrological schemes possessing moderate losses in the nearest future. At the same time, it is instructive to analyze quantum regimes with coupled solitons (see Fig. 1) established in this work. We plan a more detailed study of the quantum phase transition problem that is inherent to the LMG model and has not been verified in the paper. We will publish the analysis of these problems in the future.

## Supplementary Information

Supplementary Information.
