# Supplementary material for: Bose–Einstein condensate soliton qubit states for metrological applications
Source: Sci Rep. 2021 Sep 29;11:19363. doi: 10.1038/s41598-021-97971-4 (PMC8481417; doi:10.1038/s41598-021-97971-4)
Supplement: Supplementary file 1 — Supplementary Information. [file 41598_2021_97971_MOESM1_ESM.pdf]

Supplementary Material: Bose-Einstein condensate soliton qubit states for metrological applications

The Vinh Ngo[1], Dmitriy V. Tsarev[1], Ray-Kuang Lee[2,3,4,*], and Alexander A. Alodjants[1]

[1]ITMO University, Institute of Advanced Data Transfer Systems, St. Petersburg, 197101, Russia
[2]Institute of Photonics Technologies, National Tsing Hua University, Hsinchu, 30013,Taiwan
[3]National Center for Theoretical Sciences, Physics Division, Taipei, 10617, Taiwan
[4]Center for Quantum Technology, Hsinchu, 30013, Taiwan
[*]rklee@ee.nthu.edu.tw

ABSTRACT

Appendix: Approximation of functionals I and J.

Solutions of Eqs. (15) strictly depend on functionals I and J and their derivatives $I' \equiv dI/dz$ and $J' \equiv dJ/dz$ defined in Eq. (14). In Fig. S1, we represent I and J as two-dimension surfaces given in $z-\Delta$ plane. From Fig. S1 (a,b), it is seen that I and J approach zero for a large Δ, excluding the edge domains where $|z| \simeq 1$. As for small Δ inherent to $0 \leq \Delta < 1.5$, we apply the polynomial approximations of I and J to illustrate their numerical estimations.

In particular, within domain $0 \leq \Delta < 0.6$, $I, J(z)$ can be effectively approximated by the forth-order polynomials as follows

$$I(z,\Delta) \approx a_I(\Delta)z^4 + b_I(\Delta)z^2 + c_I(\Delta); \quad (1a)$$
$$J(z,\Delta) \approx a_J(\Delta)z^4 + b_J(\Delta)z^2 + c_J(\Delta), \quad (1b)$$

where the coefficients are the polynomials themselves:

$$a_I = -\Delta^2 - 0.52\Delta + 0.1; \quad (2a)$$
$$b_I = 2\Delta^2 + 0.76\Delta - 0.42; \quad (2b)$$
$$c_I = -1.16\Delta^2 - 0.24\Delta + 1.33; \quad (2c)$$
$$a_J = -2\Delta^2 - 0.72\Delta + 0.4; \quad (2d)$$
$$b_J = 3.9\Delta^2 + 1.03\Delta + 0.07; \quad (2e)$$
$$c_J = -1.9\Delta^2 - 0.32\Delta + 2.7. \quad (2f)$$

At $0.6 < \Delta \leq 1.5$, in Eq. (1) the sixth-order polynomial approximation is required:

$$I(z,\Delta) \approx a_I(\Delta)z^6 + b_I(\Delta)z^4 + c_I(\Delta)z^2 + d_I(\Delta); \quad (3a)$$
$$J(z,\Delta) \approx a_J(\Delta)z^6 + b_J(\Delta)z^4 + c_J(\Delta)z^2 + d_J(\Delta), \quad (3b)$$

where

$$a_I = 0.31\Delta^2 - 2.57\Delta + 1.43; \quad (4a)$$
$$b_I = 0.9\Delta^2 + 1.24\Delta - 1.6; \quad (4b)$$
$$c_I = -1.9\Delta^2 + 3.5\Delta - 0.67; \quad (4c)$$
$$d_I = 0.69\Delta^2 - 2.21\Delta + 1.85; \quad (4d)$$
$$a_J = -1.5\Delta^2 - 0.13\Delta + 0.89; \quad (4e)$$
$$b_J = 4.62\Delta^2 - 4.78\Delta + 0.15; \quad (4f)$$
$$c_J = -4\Delta^2 + 8.4\Delta - 1.45; \quad (4g)$$
$$d_J = 0.94\Delta^2 - 3.52\Delta + 3.56. \quad (4h)$$

With thees approximations, an error less than 4% for any $-1 \leq z \leq 1$ and Δ in the mentioned domains can be achieved.

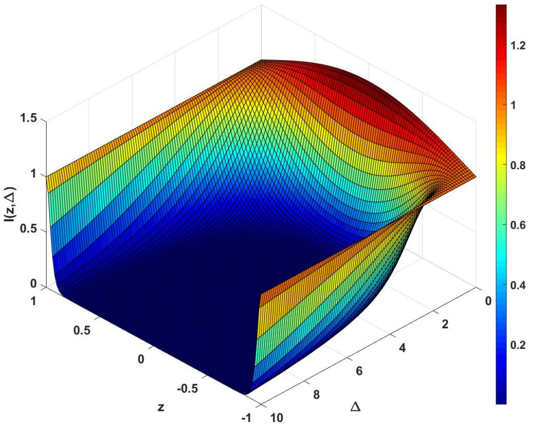

a)

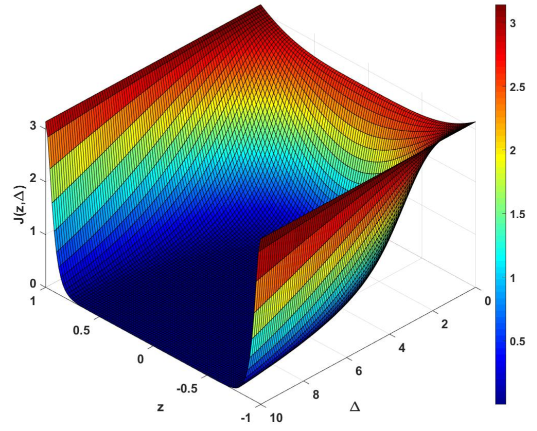

b)

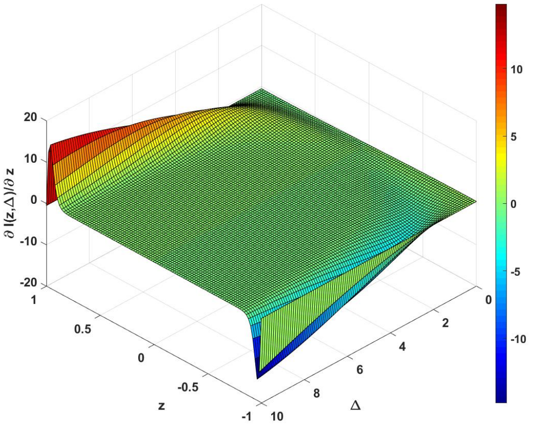

c)

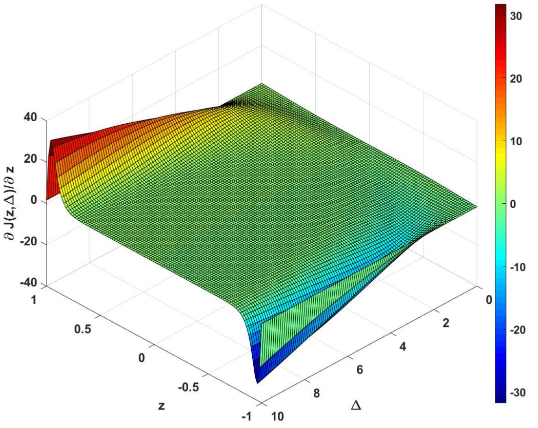

d)

Figure S1. (a-b) The I, J functionals and (c-d) their derivatives I', J', versus z and Δ.
